# Supplementary figures and images for: Determining the sample size required to establish whether a medical device is non-inferior to an external benchmark
Source: BMJ Open. 2017 Aug 28;7(8):e015397. doi: 10.1136/bmjopen-2016-015397 (PMC5652499; doi:10.1136/bmjopen-2016-015397)

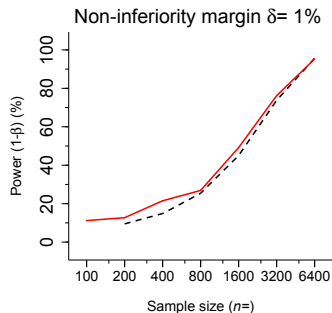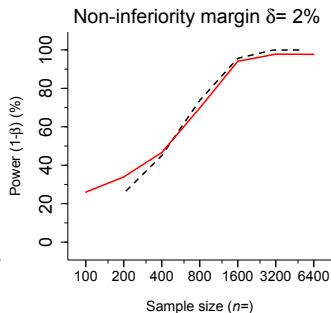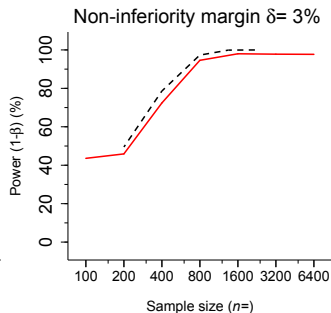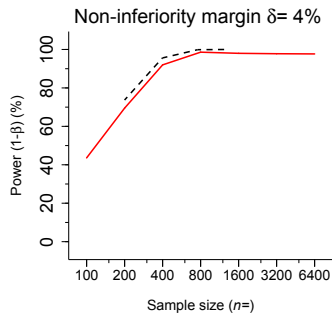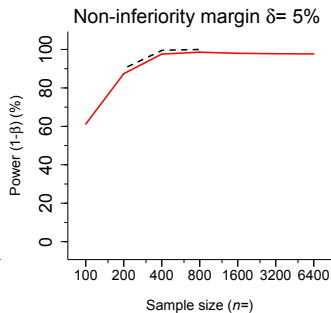

----- Analytic sample size

— Simulation sample size

Supplement: Supplementary file 1 [file bmjopen-2016-015397supp001.pdf]
