# Supplementary material for: Determining the sample size required to establish whether a medical device is non-inferior to an external benchmark
Source: BMJ Open. 2017 Aug 28;7(8):e015397. doi: 10.1136/bmjopen-2016-015397 (PMC5652499; doi:10.1136/bmjopen-2016-015397)
Supplement: Supplementary file 2 [file bmjopen-2016-015397supp002.pdf]

Supplementary Table 1. Tabulation of performance metrics of simulation results.

Method	N	Bias	RMSE	Coverage	95% CI Width	Power (1-β) to Detect Non-Inferiority (%) δ=0	δ=1	δ=2	δ=3	δ=4	δ=5
KM NCR	100	0.01	1.70	96.8	9.35	0.6	0.6	2.7	11.2	11.2	26
	200	-0.03	1.22	96.8	6.29	0.9	6.8	12.7	34	46	69.6
	400	0.01	0.88	94.3	4.36	1.8	9.9	30.2	64.6	84.3	96.1
	800	0.02	0.63	95.5	3.06	2	19.2	59.3	90.9	97.5	97.5
	1600	-0.01	0.43	96.1	2.14	1.4	40	91.3	97.5	97.5	97.5
	3200	-0.02	0.31	95.1	1.51	2.2	71.3	97.3	97.3	97.3	97.3
	6400	0.01	0.22	96.3	1.07	1.2	93.6	97.5	97.5	97.5	97.5
KM CR	100	0.03	1.90	94.9	10.08	1	1	2.3	6.6	13.9	24.4
	200	-0.02	1.32	96.1	6.76	1.2	4.9	11.7	26.1	43.6	61.6
	400	0.01	0.95	94.3	4.68	1.6	8.7	28.2	54.8	79.7	92.9
	800	0.02	0.68	94.8	3.26	2.6	17.3	54.2	86.5	97.4	97.4
	1600	-0.02	0.46	95.4	2.29	1.8	35.4	87.8	97.2	97.2	97.2
	3200	-0.02	0.32	94.9	1.61	2.1	63.6	97	97	97	97
	6400	0.00	0.23	95.2	1.14	2	90.6	97.2	97.2	97.2	97.2
CIF CR	100	-0.52	1.73	93.1	8.04	5.6	5.6	17.4	34.8	34.8	53.4
	200	-0.57	1.27	93.3	5.68	5.8	11.6	33.5	47.3	72.4	88.8
	400	-0.54	0.96	93.4	4.03	6.1	30.1	58.1	81.9	95.5	99.3
	800	-0.53	0.75	88.2	2.86	11.4	49.7	86.9	99.3	99.6	99.6
	1600	-0.57	0.65	81.2	2.02	18.4	79.4	98.9	99.6	99.6	99.6
	3200	-0.56	0.59	68.7	1.43	31.3	98	100	100	100	100
	6400	-0.55	0.55	46.1	1.01	53.9	100	100	100	100	100
Z-test NCR	100	0.01	1.70	88.4	8.17	11.2	11.2	26	43.6	43.6	61.2
	200	-0.03	1.22	92.3	5.94	6.8	12.7	33.9	45.9	69.5	87.4
	400	0.01	0.88	92	4.25	6.7	21.5	46.6	72.4	92	97.6
	800	0.02	0.63	94.2	3.02	4.4	26.9	69.8	94.6	98.6	98.6
	1600	-0.01	0.43	95.3	2.13	2.7	49.2	94.1	98	98	98
	3200	-0.02	0.31	94	1.51	3.8	76.2	97.8	97.8	97.8	97.8
	6400	0.01	0.22	95.9	1.07	1.8	95.3	97.7	97.7	97.7	97.7
Z-test CR	100	1.28	2.47	92.1	10.61	5.6	5.8	14.6	17.6	26.8	35.2
	200	1.22	1.86	94.6	7.81	1.9	5.6	11.4	21.9	33.9	49
	400	1.25	1.53	90.1	5.57	0.4	4	12.6	30.1	50.5	70.3
	800	1.27	1.38	78.7	3.96	0.1	2.9	13.3	44.2	72.9	78.8
	1600	1.21	1.23	63.2	2.80	0	1.5	21.9	63.2	63.2	63.2
	3200	1.22	1.22	31.4	1.98	0	0.9	31.4	31.4	31.4	31.4
	6400	1.25	1.25	4.8	1.40	0	0.3	4.8	4.8	4.8	4.8

KM= Kaplan-Meier ; CIF= Cumulative Incidence Function; NCR=No Competing Risks ; CR= Competing Risks ; δ =Non-inferiority margin
