# Supplementary material for: Determining the sample size required to establish whether a medical device is non-inferior to an external benchmark
Source: BMJ Open. 2017 Aug 28;7(8):e015397. doi: 10.1136/bmjopen-2016-015397 (PMC5652499; doi:10.1136/bmjopen-2016-015397)
Supplement: Author's manuscript [file bmjopen-2016-015397.draft_revisions.pdf]

BMJ Open

BMJ Open is committed to open peer review. As part of this commitment we make the peer review history of every article we publish publicly available.

When an article is published we post the peer reviewers' comments and the authors' responses online. We also post the versions of the paper that were used during peer review. These are the versions that the peer review comments apply to.

The versions of the paper that follow are the versions that were submitted during the peer review process. They are not the versions of record or the final published versions. They should not be cited or distributed as the published version of this manuscript.

BMJ Open is an open access journal and the full, final, typeset and author-corrected version of record of the manuscript is available on our site with no access controls, subscription charges or pay-per-view fees (http://bmjopen.bmj.com).

If you have any questions on BMJ Open's open peer review process please email info.bmjopen@bmj.com

BMJ Open

Determining the sample size required to establish whether a medical device is non-inferior to an external benchmark.

Journal:	*BMJ Open*
Manuscript ID	bmjopen-2016-015397
Article Type:	Research
Date Submitted by the Author:	01-Dec-2016
Complete List of Authors:	Sayers, Adrian; University of Bristol, Muscloskeletal Research Unit<br>Crowther, Michael; University of Leicester, Department of Health Sciences<br>Judge, Andrew; University of Oxford, NIHR Musculoskeletal Biomedical Research Unit<br>Whitehouse, Michael; University of Bristol, Musculoskeletal Research Unit<br>Blom, Ashley; University of Bristol, School of Clinical Sciences
**Primary Subject Heading**:	Research methods
Secondary Subject Heading:	Epidemiology
Keywords:	Benchmark, Sample size, Non-inferiority, Medical Implant, Competing Risks

SCHOLARONE™
Manuscripts

For peer review only

Determining the sample size required to establish whether a medical device is non-inferior to an external benchmark.

Adrian Sayers[1, 2], Michael J Crowther[3], Andy Judge[4, 5], Michael R Whitehouse[1], Ashely Blom[1]

1. Musculoskeletal Research Unit, School of Clinical Sciences, University of Bristol, Level 1 Learning and Research Building, Southmead Hospital, Westbury-on-Trym Bristol, BS10 5NB
2. School of Social and Community Medicine, University of Bristol, 39 Whatley Road, Bristol, BS8 2PS.
3. Biostatistics Research Group, Department of Health Sciences, College of Medicine, Biological Sciences and Psychology, University of Leicester, Centre for Medicine, University Road, Leicester, LE1 7RH, UK
4. NIHR Musculoskeletal Biomedical Research Unit, Nuffield Department of Orthopaedics, Rheumatology and Musculoskeletal Sciences, University of Oxford, Oxford, OX3 7LD
5. MRC Lifecourse Epidemiology Unit, University of Southampton, Southampton General Hospital, Southampton, SO16 6YD

Corresponding author:

Name: Adrian Sayers

Email: adrian.sayers@bristol.ac.uk Tel: +44 (0) 117 41 47880

Keywords: Arthroplasty, Benchmark, Sample size, Non-inferiority, Epidemiology, Medical Implant, Competing Risks

For peer review only - http://bmjopen.bmj.com/site/about/guidelines.xhtml

Abstract

Objectives: The use of benchmarks to assess the performance of implants such as those used in arthroplasty surgery is a widespread practice. It provides surgeons, patients and regulatory authorities with the reassurance that implants used are safe and effective. However, it is not currently clear how or how many implants should be statistically compared to a benchmark to assess whether or not that implant is superior, equivalent, non-inferior, or inferior to the performance benchmark of interest.

We aim to describe the methods and sample size required to conduct a one sample non-inferiority study of a medical device for the purposes of Benchmarking.

Design: Simulation study

Setting: National register of medical devices

Methods: We simulated data, with and without a non-informative competing risk, to represent an arthroplasty population and describe three methods of analysis (Z-test, 1- Kaplan Meier, and competing risks) commonly used in surgical research.

Primary Outcome: We evaluate the performance of each method using power, bias, and precision.

Results: 1-Kaplan Meier provides an unbiased estimate of implant failure which can be used to asses if a surgical device is non-inferior to an external benchmark. Small non-inferiority margins require significantly more individuals to be at risk compared to current benchmarking standards.

Conclusion: A non-inferiority testing paradigm provides a useful framework for determining if an implant meets the required performance defined by an external benchmark. The sample size required at the onset of the study is ultimately determined by the non-inferiority margin and the willingness to incorrectly reject non-inferior devices that are tolerable to patients, surgeons, and commissioners. It is clear, when benchmarking implant performance, net failure estimated using 1-KM is preferential to crude failure estimated by competing risk models.

For peer review only - http://bmjopen.bmj.com/site/about/guidelines.xhtml

Strengths and limitations of this study

- We propose a one-sample non-inferiority design for assessing the failure rate of medical devices against an external benchmark, using arthroplasty as an exemplar.
- Using a simulation study we demonstrate that device failure rate estimated using Kaplan Meier is appropriate, and provides unbiased estimates of implant failure in the context of benchmarking arthroplasty.
- The number of individuals required at the beginning of a study in order to obtain nominal power at 5 different non-inferiority margins is described.
- Performance of 5 methods under 2 data generating processes is described in terms of bias, Root Mean Square Error (RMSE), coverage and confidence interval width.
- We assume simple analyses using Kaplan Meier derived from population registry studies can provide causal interpretations.

Funding Statement

AS was supported by a MRC strategic skills fellowship: MRC Fellowship MR/L01226X/1

Competing interest statements

No authors have any competing interests to declare.

Author Contributions

AS conceived and designed the study, AS MC AJ MW AB interpreted the data, revised the manuscript, and approved the final draft.

Data Sharing

No data is available to be shared.

For peer review only

Introduction

Arthroplasty prosthesis are not currently required to undergo randomised clinical trials prior to their introduction into routine clinical practice, and post-market surveillance is used to determine their efficacy. Without head to head comparison against existing products, devices can be compared to external references or benchmarks. Product benchmarking in medical devices is common and is intended to help surgeons and health care administrators select safe and effective medical devices, yet there is no consensus on how this should be performed.

In arthroplasty, the process of benchmarking prosthetic implants is extensive. However, there is considerable debate with regards to the standards and criteria that should be adopted.[1] It has recently been suggested that the benchmark failure rate for prosthetic implants should become more stringent and reduced from the 10% to 5% at 10 years,[2] and the National Institute of Clinical Excellence (NICE) have incorporated this into guidance with regards to patients with end stage arthritis of the hip.[3]

Benchmarking bodies around the world were quick to respond and the ODEP (UK)[4], PLAC (Australia)[5], NOV (Netherlands)[6] bodies have set their highest benchmark to 5% failure at 10 years in total hip prostheses.[4-6] Despite subtle differences in each countries benchmarking system, a common question is how many implanted devices do we need to observe to determine if the device meets the specified benchmark or not? Both the UK and Netherlands require that there should be at least 500 implants remaining at risk at the end of the benchmarking period,[4 6] but with no clear rationale of why 500 implants is more or less suitable than 5000. Australia does not specify the numbers left at risk at the end of 10 years, but require data to be supported from their national arthroplasty registry,[5] which is one of the world's largest arthroplasty registers.

Despite the recommendations of a maximum failure rate of 5% at 10 years,[2] it is not described how the data should be compared to the proposed benchmark. Therefore, the evaluation process is somewhat ill defined, and uncertainty around the estimates are not formally included in the decision making process. Furthermore, there is ambiguity about how failure should be estimated and whether or not net or crude failure (estimands) are of primary interest.[7] Net failure is only possible in a hypothetical world of immortal patients i.e. the competing event (death) is prevented from occuring,[8] and crude failure is the real world probability of failure allowing for the competing risk (death).[7]

For peer review only - http://bmjopen.bmj.com/site/about/guidelines.xhtml

Whilst benchmarking in arthroplasty is used as an exemplar, similar problems and arguments can be made in any medical discipline which utilise medical devices or implants e.g. cardiac or cosmetic surgery.

In a simple setting of a clinical study with no loss to follow up and no censoring, if implant survival is calculated and it is less than or equal to the benchmark do we conclude that the implant has reached the benchmark? i.e. $\widehat{P_F} <= P_{BM}$, where $\widehat{P_F}$ is the proportion of failed implants and P_{BM} is the proportion of failure defined by the benchmark. It is clear that this statement does not include a description with regards to the uncertainty of the estimate. Therefore, as in the majority of the scientific literature, we would probably consider conducting a one-sample hypothesis test or (more preferably) constructing a confidence interval of whether or not the proportion of implant failures at 10 years is not greater than the benchmark plus some level of uncertainty, [9 10] i.e. H_1: $\widehat{P_F} \pm \Phi(z_{1-\alpha/2})\widehat{SE}(\widehat{P_F}) >= P_{BM}$, where $\Phi(z)$ is a centile of a standard normal distribution, and α is the Type I error rate. An analytical strategy equivalent to this would allow us to make probability statements such as "The average implant failure at 10 years is estimated to be P_F, and in repeated sampling we would expect on $1-\alpha/2$ % of occasions that the true, unknown, value is contained within the confidence interval". This approach can be framed as a one-sample hypothesis test (*z*-test), of one proportion, see equation 1.

$$z = \frac{\widehat{P_F} - P_{BM}}{\sqrt{\frac{\widehat{P_F}(1-\widehat{P_F})}{n}}} \qquad 1$$

The temptation is to simply rearrange equation 1, solve the hypothesis test for *n,* and conclude that this is the minimal sample size required to detect if an implant is superior to the benchmark. However, this would be incorrect as this fails to recognise the uncertainty introduced by sampling variability. With small samples, sampling variability is large, and conversely when samples are large sampling variability is small. Sampling variability is one of the reason that Type I and Type II statistical errors are made. A Type I error is made when we incorrectly reject a valid null hypothesis, i.e. that the true device failure rate at 10 years is equal to the benchmark. Whereas a Type II error is when the null hypothesis is incorrect, i.e. the true failure rate is not equal to the benchmark, yet we fail to reject it. One minus the Type II error rate is also known as power, see figure 1.

For peer review only - http://bmjopen.bmj.com/site/about/guidelines.xhtml

Figure 1. Statistical Error in hypothesis testing framework

		The truth	
		H_0 is True	H_0 is False
Decision with observed data	Reject H_0:	Type I Error (α)	True positives or power $(1-\beta)$
	Fail to reject H_0:	True negatives	Type II Error (β)

H_0: Device failure rate is greater than the benchmark

H_1: Device failure rate is less than or equal to the benchmark

Whilst there has been some debate with regards the use of formal sample size calculations, which are determined on the basis of power and type I errors, or by pre-specifying the desired width of the confidence interval.[11 12] We believe they are both useful tools for researchers and device manufacturers when planning clinical studies, and determining the sample sizes required to draw reliable conclusions, by explicitly stating expected power of a sample and or defining the desired width of estimated confidence interval. When planning a clinical study, decisions need to be made with regards to the uncertainty in which the null hypothesis should be rejected (commonly but not necessarily $\alpha = 0.05$), and power $(1-\beta)$ which is the ability to correctly reject the null hypothesis when it is false. Therefore, simply solving a hypothesis test for n fails to consider the power to detect an effect when it is truly present. A study with a sample size that gives 100% power will correctly reject the null hypothesis on all occasions, and correspondingly a sample size that gives 50% power will correctly reject the null hypothesis on only half of occasions. This is the essence of nearly all superiority designed clinical studies.

However, in the context of a benchmarking system the definition of superiority is restrictive. For example, if the true failure rate of the implant of interest is exactly equal to the benchmark ($\widehat{P_F} = P_{BM}$), it would be desirable to say that the implant has met the required standard. However, it is impossible to demonstrate that it is superior, because the standard error will always be positive and not 0, and therefore the upper confidence interval will always be greater than the benchmark ($\widehat{P_F} + \Phi\left(z_{1-\alpha/2}\right)\widehat{SE}\left(\widehat{P_F}\right) > P_{BM}$). Therefore, in a

For peer review only - http://bmjopen.bmj.com/site/about/guidelines.xhtml

superiority study design, if a prosthesis failure rate is truly equivalent to the benchmark (the null hypothesis is correct), in repeated sampling we should only incorrectly reject it on 5% of occasions if $\alpha = 0.05$. Therefore, when comparing devices to a benchmark, a non-inferiority study design including sample size calculations, analysis, and reporting maybe preferable.[13-17]

Despite the linguistic similarities between superiority and non-inferiority studies, the analysis and interpretation is different. A non-inferiority framework requires the interested parties to place limits around what could be described as non-inferior i.e. a non-inferiority margin (δ). This is stating that if a device failure rate was 5%, the 95% confidence interval ranged between 4.01% and 5.99%, and that the non-inferiority margin was 1%, we would be happy to conclude that device was clinically equivalent or non-inferior, see figure 2.

Figure 2: Schematic representation of inferiority, non-inferiority and superiority studies

<Figure 2 Here>

If the failure rate was 5.5% and the confidence interval ranged between 5.25% and 5.75% we would still conclude that this device was clinically non-inferior, despite being statistically inferior, with a non-inferiority margin of 1%.

The aim of this study is to investigate the sample size required at the beginning of a study to demonstrate superiority and non-inferiority of implant failure compared to a benchmark level of performance, and the consequences of using three common methods of estimating failure in a simulation study.

For peer review only

Method

The simulation study will be described using an Aims, Data generating process, Method of analysis, Estimands, and Performance (ADMEP) structure.

Aims

The aim of this study is to describe the sample size required to identify if a prosthetic implant has a failure rate non-inferior to an external bench mark using simple analytical solutions. In addition, we will use a simulation study to determine the power to detect superiority and non-inferiority with different sample sizes, different estimands, in the presence of a non-informative competing risk (CR) (mortality), and when the true implant failure rate is the same as the benchmark.

Analytical Sample Size Calculation of One Proportion

Using analytical solutions of a *z*-test in a non-inferiority setting (see equation 2 and 3), the sample size at five non-inferiority margins (δ) at 1, 2, …., 5% was calculated with power $(1-\beta)$ varied from 10% to 99.9%. The proportion of failures ($\widehat{P_F}$) was assumed to be equal to the benchmark (P_{BM}), 5% and the Type I error rate was set at α = 2.5% (the one tail equivalent of α =0.05).

$$z = \frac{\widehat{P_F} - P_{BM} - \delta}{\sqrt{\frac{\widehat{P_F}(1-\widehat{P_F})}{n}}} \quad 2$$

$$n = \widehat{P_F}(1-\widehat{P_F})\left(\frac{z_{1-\alpha} + z_{1-\beta}}{\widehat{P_F} - P_{BM} - \delta}\right)^2 \quad 3$$

Data Generating Process (DGP)

Two different data generating mechanisms with varying sample sizes (N= 100, 200, 400, 800, 1600, 3200, 6400) were explored: 1) implant failure with no censoring, and 2) implant failure with censoring for mortality. Implant failure and mortality data was simulated independently (non-informative censoring) from a parametric survival distribution (2 parameter Weibull) (Failure: $T \sim Weib(\lambda = 0.01, \gamma = 0.71)$; Mortality: $T \sim Weib(\lambda = 0.017, \gamma = 1.32)$, where λ and γ are scale and shape parameters in a Weibull distribution respectively.[18 19] Failure and

For peer review only - http://bmjopen.bmj.com/site/about/guidelines.xhtml

mortality were simulated to have 5% and 30% occurrence at ten years respectively, and administratively censored at 10 years. 1000 repetitions of each DGP were simulated.

Method of Analysis

We investigated the first DGP (no competing risks) using the z-test, see equation 2, where the proportion of failures is simply estimated by the number of observed failures (f) within the sample divided by the total sample.

$$\widehat{P_F} = \frac{f}{n} \qquad 4$$

A failure function, 1-$\hat{S}(t)$[20], approach was used to estimate failure where n_j is the number at risk of failure preceding time t_j and f_j is the number of failures at time t_j, see equation 5, with confidence intervals estimated using asymptotic variance of $log\left(-\log \hat{S}(t_j)\right)$.[21]

$$\hat{S}(t) = \prod_{j|t_{j\leq t}} \left(\frac{n_j - f_j}{n_j}\right) \qquad 5$$

The second DGP was similarly investigated using 3 approaches: 1) A z-test, where the proportion of failures was calculated excluding those who died prior to 10 years; 2) A 1-Kaplan Meier (1-KM) approach, with the addition of censoring individuals when they died was also used. 3) A non-parametric competing risk (CR) model[22] was also explored, see equation 6. The CR model calculates the cumulative incidence for failure type k. It is estimated using a KM approach for all failure types at the instance before the failure of interest, $\hat{S}(t_{j-1})$, and then multiplying it by the cause specific hazard, d_{kj}/n_j, i.e those experiencing the failure of interest divided by all individuals who remain at risk.

$$\widehat{CIF}_k(t) = \sum_{j|t_{j\leq t}} \hat{S}(t_{j-1}) \frac{d_{kj}}{n_j} \qquad 6$$

All analyses were conducted in Stata (Stata Statistical Software: Release 14.1 College Station, TX: StataCorp LP.)

For peer review only - http://bmjopen.bmj.com/site/about/guidelines.xhtml

Estimands

The estimand of interest was cumulative implant failure at 10 years, and its 95% confidence intervals, in a hypothetical world where implant failure is the only possible outcome.

The first DGP explicitly simulates data where implant failure is the only possibly outcome, and the analytical methods purport to estimate net failure, whereas the second DGP simulates data where there are two potential outcomes (implant failure or death). The simple proportion of failures ($\widehat{P_F}$) and failure function (1-$\hat{S}(t)$) both estimates net failure (assuming the untestable assumption of independence between the CR and implant failure). Whereas the CR approach ($\widehat{CIF}_k(t)$) estimates crude failure, which can be described as the real world probability of failure, which allows for the fact that some individuals die before their implant fails.

Performance

Performance was assessed in the superiority study setting using bias, root mean square error (RMSE) (absolute bias), power to detect superiority, coverage of the 95% confidence interval (1-Type I error), and confidence interval width. Similarly we estimated performance in a non-inferiority setting using power (1-Type II error) to detect non-inferiority at 1, 2, 3, 4 and 5% at 10 years.

For peer review only - http://bmjopen.bmj.com/site/about/guidelines.xhtml

Results

Analytical approach to sample size calculation of non-inferiority against a benchmark

Using analytic sample size calculations, non-inferiority against a bench mark sample size was calculated and is presented in Figure 3.

Figure 3. Sample size required to detect non-inferiority of the failure proportion with non-inferiority margins (δ) at 1, 2, 3, 4, and 5%.

<Figure 3 Here>

With a non-inferiority margin of 3% failure, power of 50%, 203 individuals are required at the beginning of the study, whereas with 90% power, 555 individuals are required at the beginning of the study. There is an approximately log-linear association between sample size and power between 50% and 90%, at all non-inferiority margins. However, sample size rapidly increases as the non-inferiority margin reduces.

Simulation approach to sample size calculation of superiority against a benchmark

Results from the simulation study using a superiority design are presented in Figure 4.

Figure 4: Performance characteristics of 5 analyses; *z*-test and Kaplan Meier when there is no competing risk (NCR), and a *z*-test, KM and CIF in the presence of competing risk (CR).

<Figure 4 Here>

The method of analysis and DGP process are indicated using 5 different line styles. It is clear that the cumulative incidence function (CIF), which estimates crude failure, estimated in the presence of CR consistently under estimates net failure by 0.5%, whereas the *z*-test, an estimate of net failure, in the presence of CR over estimates failure rates. Notably the variability (RMSE) in the estimates of all methods are similar, and as sample size increases they reduce. However, the CIF and *z*-test in the presence of CR are biased estimates of net failure in comparison to KM, and RMSE does not tend to zero with increasing sample sizes. Correspondingly, coverage of the 95% CI reduces as the sample size increases for both the CIF and *z*-test. The CIF power to detect superiority erroneously increases as a consequence of a consistent difference in the estimand (crude vs. net failure) and narrowing confidence intervals. The width of the estimated confidence intervals, across all methods, consistently

For peer review only - http://bmjopen.bmj.com/site/about/guidelines.xhtml

decreases as sample size increases. Despite their homogeneity, the width of the CI from the CIF is approximately 1% larger than that of the KM estimate in the presence of CR.

Simulation approach to sample size calculation of non-inferiority against a benchmark

Simulation results using a *Z*-test with no competing risks were compared with analytic sample size calculations assuming no competing risks, see supplementary figure 1. Simulation results are generally concordant with analytical results. However, analytical results tend to slightly over-estimate power when the non-inferiority margin is greater than 3%.

Results from the simulation study using a non-inferiority paradigm, are presented in Figure 5.

Figure 5: Power to detect non-inferiority at 1,2,3,4 and 5% below a 95% benchmark performance. The DGP and method of analysis are presented in separate panels. Sample size is indicated on the horizontal axis.

<Figure 5 Here>

When no competing risk is present, a 3% non-inferiority margin, and at samples sizes of 200 or 800, a *z*-test has 46% and 94% power to detect non-inferiority respectively, and KM has 34% and 91% power to detect non-inferiority. When a non-informative competing risk is present, a 3% non-inferiority margin, and sample sizes N=200 or N=800, a *z*-test has 22% & 44% power to detect non-inferiority respectively, KM has 26% and 86% power to detect non-inferiority respectively, and the CIF has 48%, and 99% power to detect non-inferiority respectively.

Mean performance estimates from the simulation study are tabulated in the supplementary table 1.

For peer review only

Discussion

This study investigates how, and how many individuals are required to demonstrate non-inferiority in failure rate of a medical device (arthroplasty prosthesis) compared to an external benchmark in the presence or absence of a competing risk.

Net failure estimated using 1-KM provides unbiased estimates in the presence or absence of a non-informative competing risk, whereas a simple Z-test or CIF over and under estimate failure in the presence of a non-informative competing risk respectively. Whilst there is reasonable agreement between analytical and simulation estimates of the sample size required to conduct a non-inferiority study, the failure to incorporate an adjustment for censoring due to mortality lead to erroneous estimates of power.

Using 1-KM to estimate failure in the presence of a non-informative competing risk (estimating net failure), a sample size of N=1600, will ensure coverage at nominal levels, have a confidence interval width of approximately 2.3% and a RMSE of 0.46%, but have a 35%, 88%, 97%, 97%, and 97% power of demonstrating non-inferiority at margins of 1%, 2%, 3%, 4%, and 5% respectively.

This study has a number of strengths. We have shown the well-known differences between net and crude failure. Despite the persistent suggestion in arthroplasty research that 1-KM over estimates failure, it is clear that it is an unbiased estimate of net failure. The CIF (crude failure) in a CR model unsurprisingly underestimates net failure, assuming independence between the CR and implant failure. We have demonstrated the number of individuals required at the onset of a benchmarking study for a variety of non-inferiority margins, under two different data generating process, and the width of the estimated CI. Despite the exemplar of arthroplasty, similar methods would apply to any discipline interested in conducting a formal benchmarking process.

However, this study has a number of important limitations. 1) We simulated data from a Weibull model with an uncorrelated competing risk, and whilst it provides a convenient and sensible method of generating data in an arthroplasty example, more complex models with correlated competing risks may be appropriate in other areas. 2) We assume that time to revision surgery is a reasonable outcome of interest. However, alternative outcomes, e.g. Patient Reported Outcome Measures (PROMS), could similarly be incorporated into a non-inferiority benchmarking design. 3) The threshold for revision is homogenous between different surgeons. 4) We have not considered the effect of analysing data from multiple

For peer review only - http://bmjopen.bmj.com/site/about/guidelines.xhtml

sources, or combining pre-existing data. However, methods of meta-analysis in a non-inferiority settings are well documented.[23 24] 5) The continuous accrual of data, and regular assessment of data against benchmarks of interest will inevitably lead to multiple hypothesis testing, and therefore the risk of incorrectly accepting or rejecting the null hypothesis of inferiority against the benchmark will increase. The decision to repeatedly look at the data must be made *a priori,* and due consideration for the multiple testing should be made in order to preserve the Type I error rate.[25] 6) Current implant rating organisations consider the failure rates of individual components (i.e. head, stem, and cups separately) opposed to the implanted construct. Despite the technical difficulties of disentangling the failures of each individual component, the multiple testing that ensues will ultimately require larger samples in order to preserve the Type I error rate. 7) We have adopted a conservative scenario, where the implant failure rate is equal to the benchmark, in a more optimistic scenario, where the implant failure rate is less than the benchmark, less individuals will be required to detect non-inferiority. Conversely, when implant failure rate is more than the benchmark, but less than the non-inferiority margin, larger samples will be required. However, unless implant manufacturers are, *a priori,* willing to propose superiority opposed to non-inferiority a conservative standpoint would appear sensible. Finally, 8) we critically assume a simple unadjusted analysis provides a causal interpretation, and there is no residual confounding or selection bias that inhibit making a fair comparison.

Despite some authors suggesting that a 1-KM over estimates implant survival in the presence of competing risk,[26] it is clear that this is not universally true, and it is very important to understand what is being estimated (net or crude failure) and for what purpose, recognise that implant failure rate is not necessarily equal to the rate of revision surgery in arthroplasty, and ensure analyses reflect the research question being presented.[27] The three methods of analysis, i.e. a *z*-test (simple proportion), 1-KM and the CIF can provide equivalent or subtly different estimates depending on the data generating process. When data are fully complete (DGP1), i.e. everyone is fully observed for the period of interest and there is no censoring, the failure rate estimated by a simple proportion, 1-KM and the CIF will all be identical, variability in coverage and power will be due to inherent differences in the methods used to construct confidence intervals. When there is censoring due to a competing risk (i.e. DGP2) differences in the estimates arise. These differences are best understood when data are fully observed for individuals experiencing the event of interest or the relevant competing event. A simple proportion (*z*-test) excludes patients who experience the competing event from the

For peer review only - http://bmjopen.bmj.com/site/about/guidelines.xhtml

denominator, whereas the CIF includes them, the numerator for both methods is simply the observed number of failures at 10 years. In this scenario it is easy to see that failure rate estimated by a simple proportion will be greater than the CIF due to the reduction in the denominator. It is also clear the CIF does not estimate the proportion of failures that would have occurred in those individuals who have experienced the competing event, and therefore will underestimate the net failure rate of the device (the numerator is too small relative to the denominator). Whereas, the KM method excludes individuals from the analysis who experience the competing risk, thereby reducing the denominator, which results in a higher failure rate than the CIF.

This balancing act is very well known, and demonstrated by the seminal work of Gooley et al.[28]. They generally recommend that researchers report the CIF, but similarly note that if one is interested in evaluating a cause specific failure the CIF may be misleading, and inferences should be made from functions which are based solely on the hazard of failure from the cause of interest, i.e. use the KM estimator. Putter et al.[8] similarly state that the "naive Kaplan–Meier estimator describes what would happen if the competing event could be prevented to occur, creating an imaginary world in which an individual remains at risk of failure from the event of interest" i.e. an immortal patient cohort. Ranstam et al.[27] describe this in an arthroplasty setting as the "implicit assumption that the patient will be alive until the implant fails". This is not to say the CIF (crude failure) is not of interest to arthroplasty surgeons, patients, regulators or healthcare planners. It is just to say the CIF (crude failure) estimates a different quantity, which reflects the real word (observed) probability of implant failure (arthroplasty revision rate), whilst acknowledging the CR of mortality explicitly prevents failure from the cause of interest. If the CIF was used in a benchmarking setting, this may have a number of different consequences: 1) it could lead to a number of implants being claimed as non-inferior to the benchmark, when they are in fact inferior, due to differences between crude and net failure, and 2) the method of estimation may lead to the selective introduction of implants in older patients where mortality is higher, which is likely to reduce the reported failure rate.

The broad success of arthroplasty, and currently used benchmark failure rate of 5% at 10 years, necessitates the use of small non-inferiority margins. A small non-inferiority margin of 1% in absolute risk represents a 20% increase in relative risk of failure compared to a benchmark of 5%. The minimum numbers of individuals required to demonstrate non-inferiority of a device where its true failure rate is equal to the benchmark is unsurprisingly

For peer review only - http://bmjopen.bmj.com/site/about/guidelines.xhtml

large. Modest sample sizes (n=1600) have limited power (35%) to detect non-inferiority, and only when sample sizes become large (n=6400) does power increase substantially (90%).

Despite differences in the performance of estimators and interpretation of estimands, the choice of sample size at the beginning of a study should be based on the desire to obtain sufficiently precise estimates (small RMSE and narrow CI's) which mitigate Type II errors for a given non-inferiority margin which is tolerable to the public, surgeons, and regulators. Whereas, the choice of sample size that should remain at risk at the end of the bench marking period is somewhat more difficult to determine. Two possible reasons to ensure the numbers at risk at the end of the period are large include; 1) maintaining the performance and minimising the width of confidence intervals and 2) ensuring sufficient numbers of patients experience 10 years of risk of revision for the estimates and the benchmarking process to be credible.

From a conservative perspective, it is simple to request that the same number of patients for a given level of power, $(1-\beta)$, and non-inferiority margin, δ, at the beginning of the benchmarking period, remain at risk at the end of the benchmarking period. However, this ignores that censoring due to mortality is expected to occur in an elderly populations, and will delay the benchmarking process. Whereas, it would not be difficult to request a specified delay (i.e. 10 years) from surgery on the jth patient until the benchmarking assessment is made, a process which is similar to pre-registration of randomised trials. This will implicitly allow for censoring due to mortality, without pre-specifying how much censoring will occur, and assumes there is no loss to follow-up (which is often assumed in arthroplasty registers).

Conclusion

The choice of non-inferiority margin, initial sample size, or desired width of the confidence interval in a benchmarking study are all subjective decisions, and can only be chosen by balancing the risk of incorrectly awarding a benchmarking standard to an implant with a failure rate beyond the non-inferiority margin, vs benchmark inflation where all devices receive benchmarks and the entire process lacks credibility. However, this study clearly demonstrates how 1-KM provides unbiased estimates of net implant failure, in a conservative scenario when the failure rate of an implant being tested is equal to the benchmark, and has a competing risk which is uncorrelated to the event of interest.

For peer review only - http://bmjopen.bmj.com/site/about/guidelines.xhtml

For peer review only

For peer review only - http://bmjopen.bmj.com/site/about/guidelines.xhtml

References

1. View of a global benchmarking system and International Working group progress. International Society of Athroplasty Registries; 2016; Manchester.
2. Kandala NB, Connock M, Pulikottil-Jacob R, et al. Setting benchmark revision rates for total hip replacement: analysis of registry evidence. *BMJ* 2015;350:h756. doi: 10.1136/bmj.h756
3. National Institue of Clinical Excelence. Total hip replacement and resurfacing arthroplasty for end-stage arthritis of the hip, 2014.
4. NHS Supply Chain. Orthopaedic Device Evaluation Panel 2016 [Available from: https://www.supplychain.nhs.uk/odep/.
5. Australian Goverment Department of Health. Prostheses List - Guide to listing and setting benefits for prostheses 2015 [Available from: http://www.health.gov.au/internet/main/publishing.nsf/Content/health-privatehealth-prostheseslist.htm.
6. Nederlandse Orthopaedische Vereniging. Classification Orthopaedic Implants: NOV Total Hip Prosthesis 2016

2016 [Available from: http://www.orthopeden.org/uploads/ky/In/kyInjf44QX2oW1qhk8PKRA/HIPLIST-NOV-2016.pdf.
7. Lambert PC, Dickman PW, Nelson CP, et al. Estimating the crude probability of death due to cancer and other causes using relative survival models. *Stat Med* 2010;29(7-8):885-95. doi: 10.1002/sim.3762
8. Putter H, Fiocco M, Geskus RB. Tutorial in biostatistics: competing risks and multi-state models. *Stat Med* 2007;26(11):2389-430. doi: 10.1002/sim.2712
9. Gardner MJ, Altman DG. Confidence-Intervals Rather Than P-Values - Estimation Rather Than Hypothesis-Testing. *Brit Med J* 1986;292(6522):746-50.
10. Sterne JA, Davey Smith G. Sifting the evidence-what's wrong with significance tests? *BMJ* 2001;322(7280):226-31.
11. Bland JM. The tyranny of power: is there a better way to calculate sample size? *BMJ* 2009;339:b3985. doi: 10.1136/bmj.b3985
12. Metcalfe C. Statistical power: still an essential element in sample size calculation. Response to:The tyranny of power: is there a better way to calculate sample size? *BMJ* 2010;339:b3985. doi: 10.1136/bmj.b3985
13. D'Agostino RB, Massaro JM, Sullivan LM. Non-inferiority trials: design concepts and issues - the encounters of academic consultants in statistics. *Stat Med* 2003;22(2):169-86. doi: 10.1002/sim.1425
14. Fleming TR. Current issues in non-inferiority trials. *Stat Med* 2008;27(3):317-32. doi: 10.1002/sim.2855
15. Koch GG. Comments on 'Current issues in non-inferiority trials' by Thomas R. Fleming, Statistics in Medicine, DOI: 10.1002/sim.2855. *Stat Med* 2008;27(3):333-42. doi: 10.1002/sim.2923
16. Sedgwick P. What is a non-inferiority trial? *Bmj-Brit Med J* 2013;347 doi: ARTN f6853

10.1136/bmj.f6853
17. Piaggio G, Elbourne DR, Pocock SJ, et al. Reporting of noninferiority and equivalence randomized trials: extension of the CONSORT 2010 statement. *JAMA* 2012;308(24):2594-604. doi: 10.1001/jama.2012.87802

For peer review only - http://bmjopen.bmj.com/site/about/guidelines.xhtml

18. Crowther MJ, Lambert PC. Simulating complex survival data. *Stata J* 2012;12(4):674-87.
19. Crowther MJ, Lambert PC. Simulating biologically plausible complex survival data. *Stat Med* 2013;32(23):4118-34. doi: 10.1002/sim.5823
20. Kaplan EL, Meier P. Nonparametric-Estimation from Incomplete Observations. *J Am Stat Assoc* 1958;53(282):457-81. doi: Doi 10.2307/2281868
21. Kalbfleisch JD, Prentice RL. The Statistical Analysis of Failure Time Data. 2nd ed ed. New York: Wiley 2002.
22. Coviello V, Boggess M. Cumulative incidence estimation in the presence of competing risks. *The Stata Journal* 2004;4(2):103-11.
23. Crowther MJ, Langan D, Sutton AJ. Graphical augmentations to the funnel plot to assess the impact of a new study on an existing meta-analysis. *Stata J* 2012;12(4):605-22.
24. Sutton AJ, Cooper NJ, Jones DR, et al. Evidence-based sample size calculations based upon updated meta-analysis. *Stat Med* 2007;26(12):2479-500. doi: 10.1002/sim.2704
25. Pocock SJ. Clinical Trials: A practical approach. Chichester: John Wiley & Sons 1983:147-154.
26. Fennema P, Lubsen J. Survival analysis in total joint replacement: an alternative method of accounting for the presence of competing risk. *J Bone Joint Surg Br* 2010;92(5):701-6. doi: 10.1302/0301-620X.92B5.23470
27. Ranstam J, Karrholm J, Pulkkinen P, et al. Statistical analysis of arthroplasty data. II. Guidelines. *Acta Orthop* 2011;82(3):258-67. doi: 10.3109/17453674.2011.588863
28. Gooley TA, Leisenring W, Crowley J, et al. Estimation of failure probabilities in the presence of competing risks: new representations of old estimators. *Stat Med* 1999;18(6):695-706.

For peer review only

For peer review only - http://bmjopen.bmj.com/site/about/guidelines.xhtml

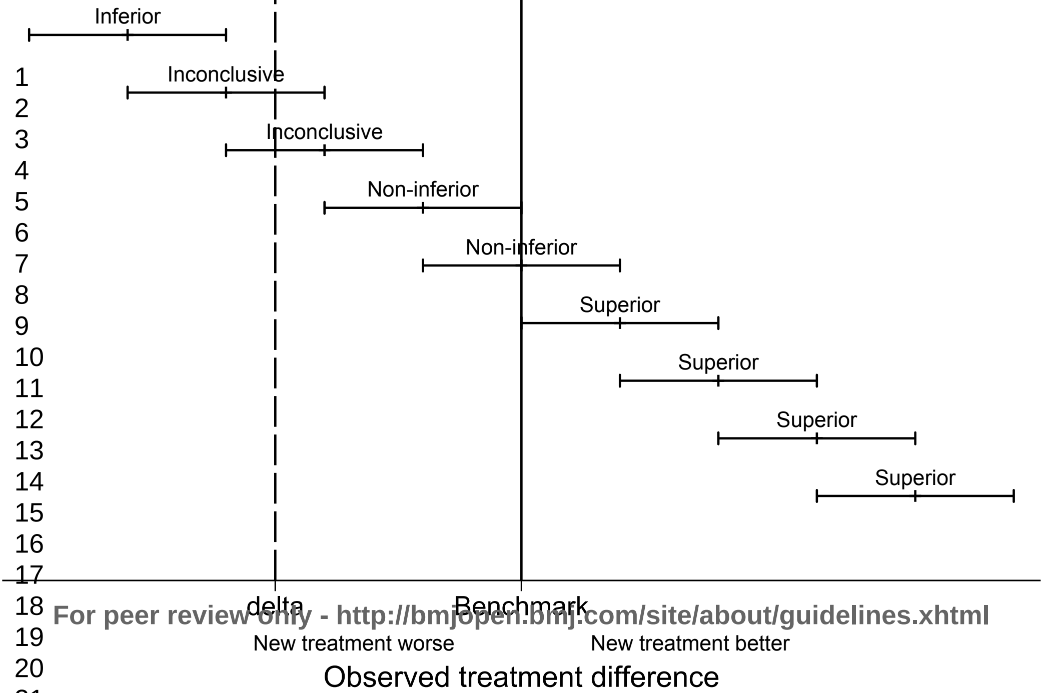

For peer review only - http://bmjopen.bmj.com/site/about/guidelines.xhtml

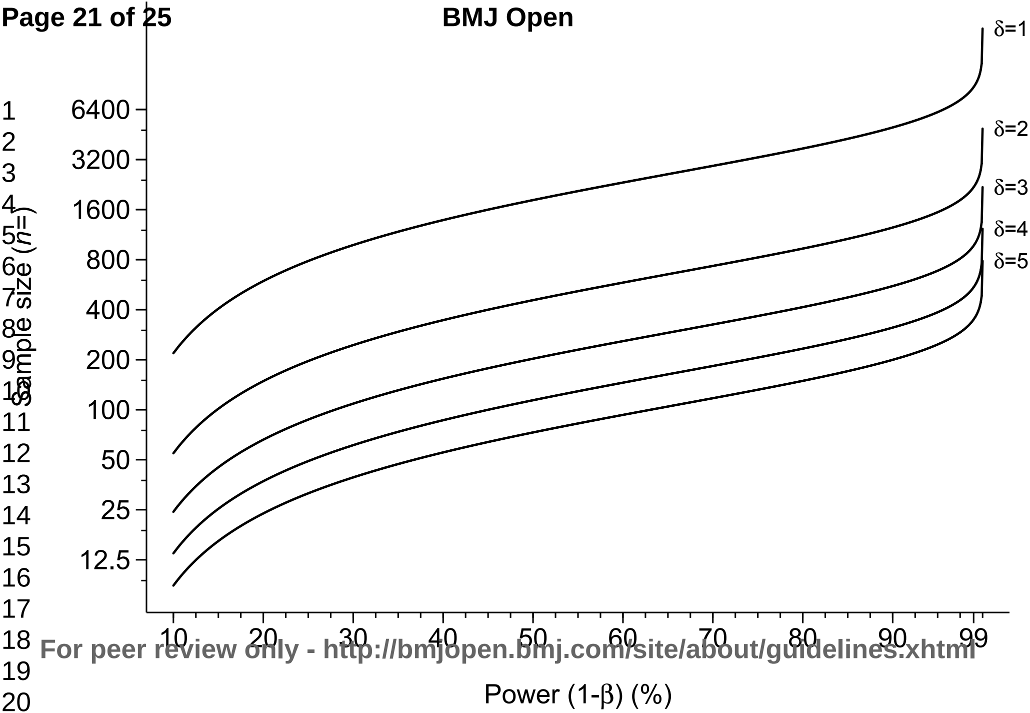

For peer review only - http://bmjopen.bmj.com/site/about/guidelines.xhtml

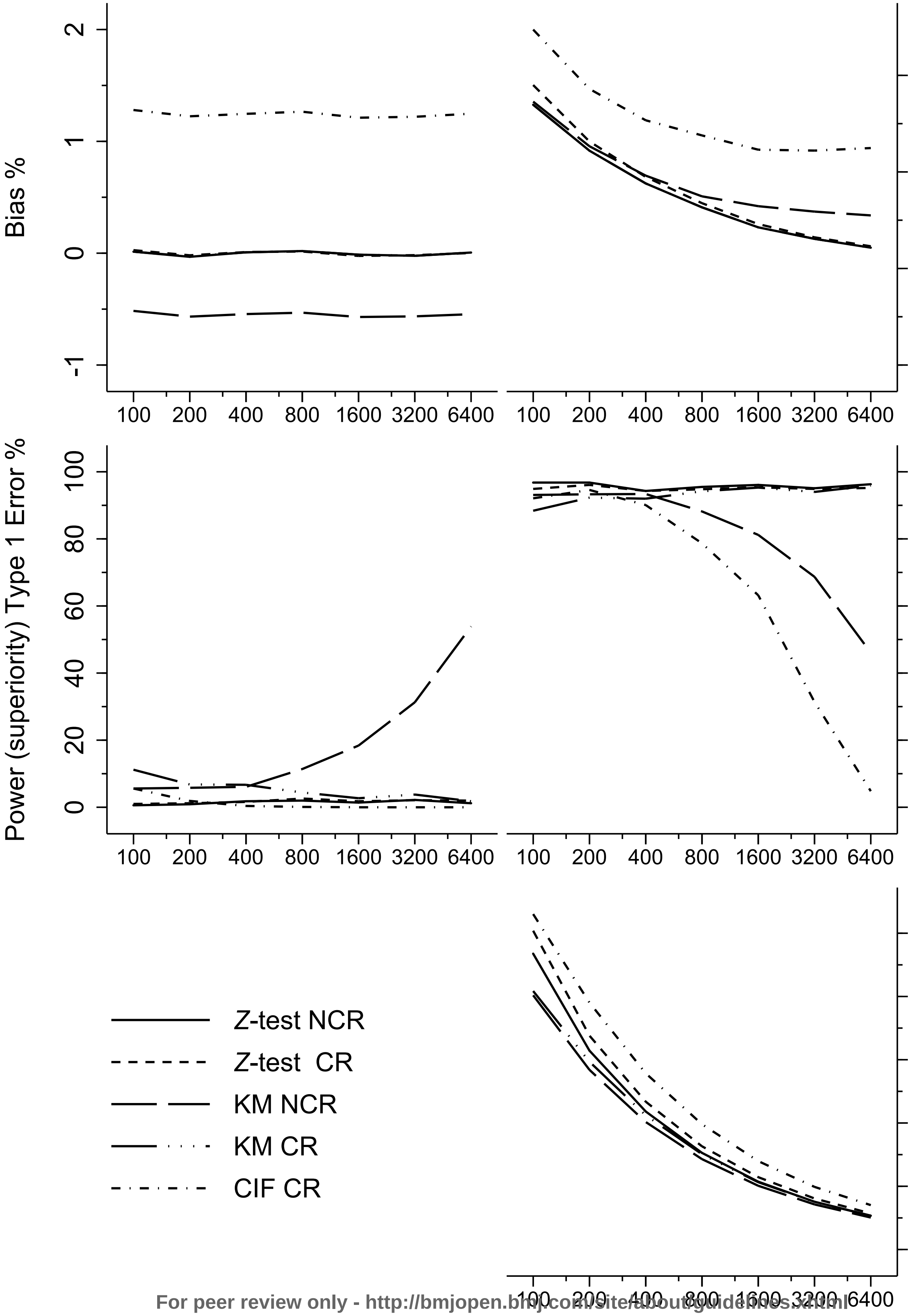
Bias %
RMSE %
Power (superiority) Type 1 Error %
Coverage %
CI width %
100 200 400 800 1600 3200 6400
Z-test NCR
Z-test CR
KM NCR
KM CR
CIF CR

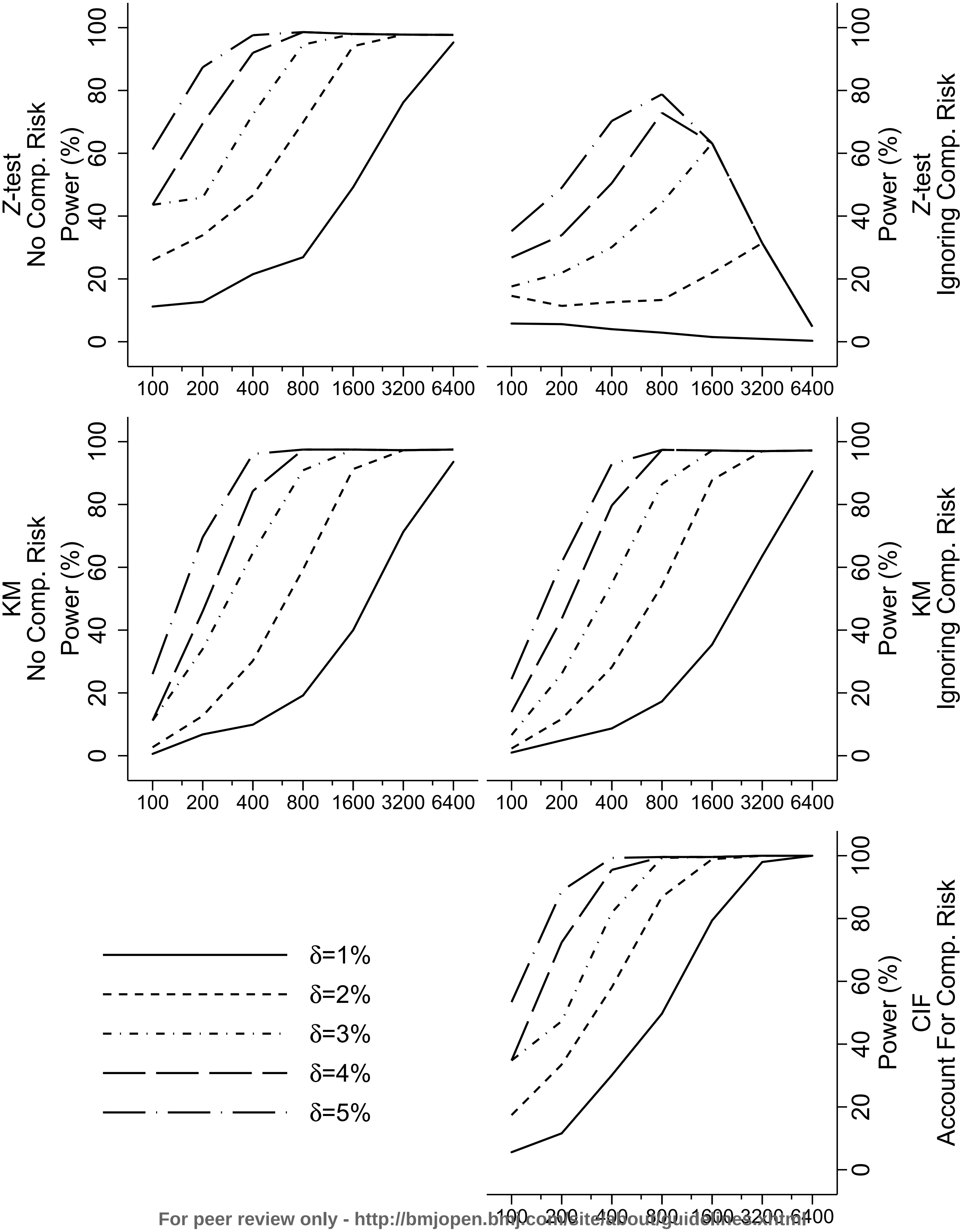
Z-test
No Comp. Risk
Power (%)
Z-test
Ignoring Comp. Risk
KM
No Comp. Risk
KM
Ignoring Comp. Risk
CIF
Account For Comp. Risk
100 200 400 800 1600 3200 6400
0 20 40 60 80 100
δ=1%
δ=2%
δ=3%
δ=4%
δ=5%

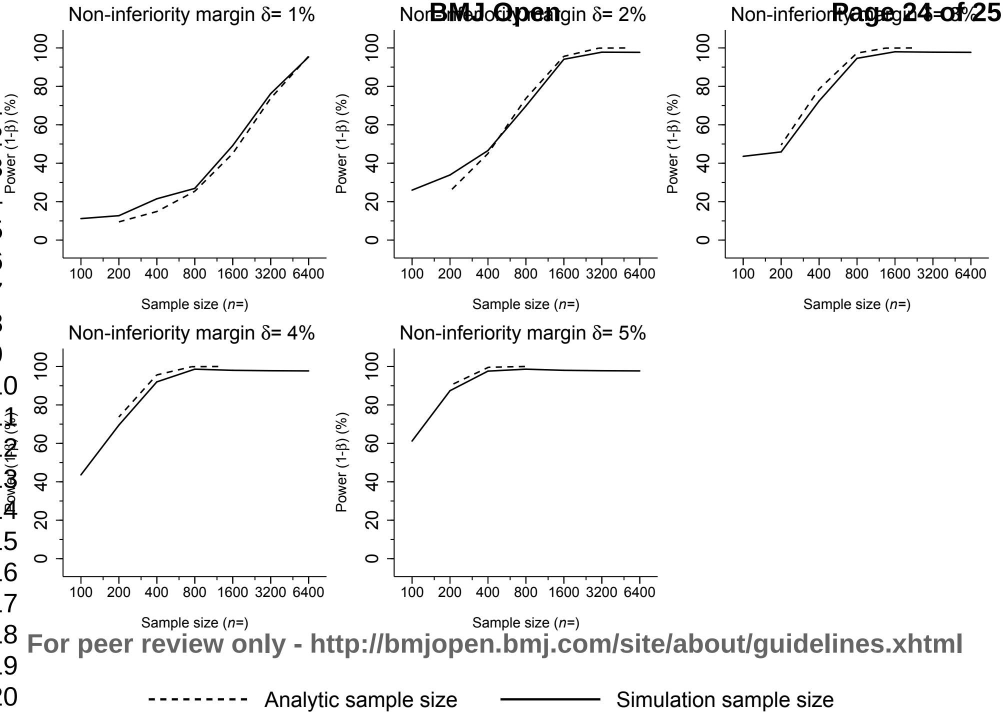

For peer review only - http://bmjopen.bmj.com/site/about/guidelines.xhtml

Supplementary Table 1. Tabulation of performance metrics of simulation results.

Method	N	Bias	RMSE	Coverage	95% CI Width	Type I Error (α)	Power (1-β) to Detect Non-Inferiority (%)				
							δ=1	δ=2	δ=3	δ=4	δ=5
KM NCR	100	0.01	1.70	96.8	9.35	0.6	0.6	2.7	11.2	11.2	26
	200	-0.03	1.22	96.8	6.29	0.9	6.8	12.7	34	46	69.6
	400	0.01	0.88	94.3	4.36	1.8	9.9	30.2	64.6	84.3	96.1
	800	0.02	0.63	95.5	3.06	2	19.2	59.3	90.9	97.5	97.5
	1600	-0.01	0.43	96.1	2.14	1.4	40	91.3	97.5	97.5	97.5
	3200	-0.02	0.31	95.1	1.51	2.2	71.3	97.3	97.3	97.3	97.3
	6400	0.01	0.22	96.3	1.07	1.2	93.6	97.5	97.5	97.5	97.5
KM CR	100	0.03	1.90	94.9	10.08	1	1	2.3	6.6	13.9	24.4
	200	-0.02	1.32	96.1	6.76	1.2	4.9	11.7	26.1	43.6	61.6
	400	0.01	0.95	94.3	4.68	1.6	8.7	28.2	54.8	79.7	92.9
	800	0.02	0.68	94.8	3.26	2.6	17.3	54.2	86.5	97.4	97.4
	1600	-0.02	0.46	95.4	2.29	1.8	35.4	87.8	97.2	97.2	97.2
	3200	-0.02	0.32	94.9	1.61	2.1	63.6	97	97	97	97
	6400	0.00	0.23	95.2	1.14	2	90.6	97.2	97.2	97.2	97.2
CIF CR	100	-0.52	1.73	93.1	8.04	5.6	5.6	17.4	34.8	34.8	53.4
	200	-0.57	1.27	93.3	5.68	5.8	11.6	33.5	47.3	72.4	88.8
	400	-0.54	0.96	93.4	4.03	6.1	30.1	58.1	81.9	95.5	99.3
	800	-0.53	0.75	88.2	2.86	11.4	49.7	86.9	99.3	99.6	99.6
	1600	-0.57	0.65	81.2	2.02	18.4	79.4	98.9	99.6	99.6	99.6
	3200	-0.56	0.59	68.7	1.43	31.3	98	100	100	100	100
	6400	-0.55	0.55	46.1	1.01	53.9	100	100	100	100	100
Z-test NCR	100	0.01	1.70	88.4	8.17	11.2	11.2	26	43.6	43.6	61.2
	200	-0.03	1.22	92.3	5.94	6.8	12.7	33.9	45.9	69.5	87.4
	400	0.01	0.88	92	4.25	6.7	21.5	46.6	72.4	92	97.6
	800	0.02	0.63	94.2	3.02	4.4	26.9	69.8	94.6	98.6	98.6
	1600	-0.01	0.43	95.3	2.13	2.7	49.2	94.1	98	98	98
	3200	-0.02	0.31	94	1.51	3.8	76.2	97.8	97.8	97.8	97.8
	6400	0.01	0.22	95.9	1.07	1.8	95.3	97.7	97.7	97.7	97.7
Z-test CR	100	1.28	2.47	92.1	10.61	5.6	5.8	14.6	17.6	26.8	35.2
	200	1.22	1.86	94.6	7.81	1.9	5.6	11.4	21.9	33.9	49
	400	1.25	1.53	90.1	5.57	0.4	4	12.6	30.1	50.5	70.3
	800	1.27	1.38	78.7	3.96	0.1	2.9	13.3	44.2	72.9	78.8
	1600	1.21	1.23	63.2	2.80	0	1.5	21.9	63.2	63.2	63.2
	3200	1.22	1.22	31.4	1.98	0	0.9	31.4	31.4	31.4	31.4
	6400	1.25	1.25	4.8	1.40	0	0.3	4.8	4.8	4.8	4.8

For peer review only

BMJ Open

Determining the sample size required to establish whether a medical device is non-inferior to an external benchmark.

Journal:	*BMJ Open*
Manuscript ID	bmjopen-2016-015397.R1
Article Type:	Research
Date Submitted by the Author:	29-May-2017
Complete List of Authors:	Sayers, Adrian; University of Bristol, Muscloskeletal Research Unit<br>Crowther, Michael; University of Leicester, Department of Health Sciences<br>Judge, Andrew; University of Oxford, NIHR Musculoskeletal Biomedical Research Unit<br>Whitehouse, Michael; University of Bristol, Musculoskeletal Research Unit<br>Blom, Ashley; University of Bristol, School of Clinical Sciences
**Primary Subject Heading**:	Research methods
Secondary Subject Heading:	Epidemiology
Keywords:	Benchmark, Sample size, Non-inferiority, Medical Implant, Competing Risks

SCHOLARONE™
Manuscripts

For peer review only

Determining the sample size required to establish whether a medical device is non-inferior to an external benchmark.

Adrian Sayers[1, 2], Michael J Crowther[3], Andy Judge[4, 5], Michael R Whitehouse[1], Ashley Blom[1]

1. Musculoskeletal Research Unit, School of Clinical Sciences, University of Bristol, Level 1 Learning and Research Building, Southmead Hospital, Westbury-on-Trym Bristol, BS10 5NB
2. School of Social and Community Medicine, University of Bristol, 39 Whatley Road, Bristol, BS8 2PS.
3. Biostatistics Research Group, Department of Health Sciences, College of Medicine, Biological Sciences and Psychology, University of Leicester, Centre for Medicine, University Road, Leicester, LE1 7RH, UK
4. NIHR Musculoskeletal Biomedical Research Unit, Nuffield Department of Orthopaedics, Rheumatology and Musculoskeletal Sciences, University of Oxford, Oxford, OX3 7LD
5. MRC Lifecourse Epidemiology Unit, University of Southampton, Southampton General Hospital, Southampton, SO16 6YD

Corresponding author:

Name: Adrian Sayers

Email: adrian.sayers@bristol.ac.uk Tel: +44 (0) 117 41 47880

Keywords: Arthroplasty, Benchmark, Sample size, Non-inferiority, Epidemiology, Medical Implant, Competing Risks

For peer review only

Abstract

Objectives: The use of benchmarks to assess the performance of implants such as those used in arthroplasty surgery is a widespread practice. It provides surgeons, patients and regulatory authorities with the reassurance that implants used are safe and effective. However, it is not currently clear how or how many implants should be statistically compared to a benchmark to assess whether or not that implant is superior, equivalent, non-inferior, or inferior to the performance benchmark of interest.

We aim to describe the methods and sample size required to conduct a one-sample non-inferiority study of a medical device for the purposes of Benchmarking.

Design: Simulation study

Setting: Simulation study of a national register of medical devices

Methods: We simulated data, with and without a non-informative competing risk, to represent an arthroplasty population and describe three methods of analysis (Z-test, 1- Kaplan-Meier, and competing risks) commonly used in surgical research.

Primary Outcome: We evaluate the performance of each method using power, bias, RMSE, coverage and confidence interval width.

Results: 1-Kaplan Meier provides an unbiased estimate of implant net failure which can be used to assess if a surgical device is non-inferior to an external benchmark. Small non-inferiority margins require significantly more individuals to be at risk compared to current benchmarking standards.

Conclusion: A non-inferiority testing paradigm provides a useful framework for determining if an implant meets the required performance defined by an external benchmark. Current contemporary benchmarking standards have limited power to detect non-inferiority and substantially larger samples sizes, in excess of 3200 procedures are required to achieve a power greater than 60%. It is clear when benchmarking implant performance, net failure estimated using 1-KM is preferential to crude failure estimated by competing risk models.

For peer review only - http://bmjopen.bmj.com/site/about/guidelines.xhtml

Strengths and limitations of this study

- We propose a one-sample non-inferiority design for assessing the failure rate of medical devices against an external benchmark, using arthroplasty as an exemplar.
- Using a simulation study we demonstrate that device failure rate estimated using Kaplan-Meier is appropriate, and provides unbiased estimates of implant failure in the context of benchmarking arthroplasty.
- The number of individuals required at the beginning of a study in order to obtain nominal power at 5 different non-inferiority margins is described.
- The performance of 3 methods under 2 data generating processes is described in terms of bias, Root Mean Square Error (RMSE), coverage and confidence interval width.
- We assume simple analyses using Kaplan-Meier derived from population registry studies can provide causal interpretations.

Funding Statement

AS was supported by a MRC strategic skills fellowship: MRC Fellowship MR/L01226X/1

Competing interest statements

No authors have any competing interests to declare.

Author Contributions

AS conceived and designed the study, AS MC AJ MW AB interpreted the data, revised the manuscript, and approved the final draft.

Data Sharing

No data is available to be shared.

For peer review only

Introduction

Arthroplasty prosthesis are not currently required to undergo randomised clinical trials prior to their introduction into routine clinical practice, and post-market surveillance is used to determine their efficacy. Without a head to head comparison against existing products, devices can be compared to external references or benchmarks. Product benchmarking in medical devices is common and is intended to help surgeons and health care administrators select safe and effective medical devices, yet there is no consensus on how this should be performed.

In arthroplasty, the process of benchmarking prosthetic implants is extensive. However, there is considerable debate with regards to the standards and criteria that should be adopted.[1] It has recently been suggested that the benchmark failure rate for prosthetic implants should become more stringent and reduced from the 10% to 5% at 10 years,[2] and the National Institute for Health and Care Excellence (NICE) have incorporated this into guidance with regards to patients with end stage arthritis of the hip.[3]

Benchmarking bodies around the world were quick to respond and the ODEP (UK)[4], PLAC (Australia)[5], NOV (Netherlands)[6] bodies have set their highest benchmark to 5% failure at 10 years in total hip prostheses.[4-6] Despite subtle differences in each countries benchmarking system, a common question is how many implanted devices do we need to observe to determine if the device meets the specified benchmark or not? Both the UK and Netherlands require that there should be at least 500 implants remaining at risk at the end of the benchmarking period,[4 6] but with no clear rationale of why 500 implants are more or less suitable than 5000. Australia does not specify the numbers left at risk at the end of 10 years, but require data to be supported from their national arthroplasty registry,[5] which is one of the world's largest arthroplasty registers.

Despite the recommendations of a maximum failure rate of 5% at 10 years,[2] it is not described how the data should be compared to the proposed benchmark. Therefore, the evaluation process is somewhat ill-defined, and uncertainty around the estimates are not formally included in the decision-making process. Furthermore, there is ambiguity about how failure should be estimated and whether or not net or crude failure (estimands) are of primary interest, with some authors recommend crude failure in implant survival studies.[7] Net failure is only possible in a hypothetical world of immortal patients i.e. the competing event (death)

For peer review only - http://bmjopen.bmj.com/site/about/guidelines.xhtml

is prevented from occurring,[8] and crude failure is the real world probability of failure allowing for the competing risk (death).[9]

Whilst benchmarking in arthroplasty is used as an exemplar, similar problems and arguments can be made in any medical discipline which utilises medical devices or implants e.g. cardiac or cosmetic surgery.

Hypothesis tests and power

In a simple setting of a clinical study with no loss to follow-up and no censoring, if implant survival is calculated and it is less than or equal to the benchmark do we conclude that the implant has reached the benchmark? i.e. $\widehat{P_F} <= P_{BM}$, where $\widehat{P_F}$ is the proportion of failed implants and P_{BM} is the proportion of failure defined by the benchmark. It is clear that this statement does not include a description with regards to the uncertainty of the estimate. Therefore, as in the majority of the scientific literature, we would probably consider conducting a one-sample hypothesis test or (more preferably) constructing a confidence interval of whether or not the proportion of implant failures at 10 years is not greater than the benchmark plus some level of uncertainty, [10 11] i.e. H_1: $\widehat{P_F} \pm \Phi(z_{1-\alpha/2})\widehat{SE}(\widehat{P_F}) >= P_{BM}$, where $\Phi(z)$ is a centile of a standard normal distribution, and α is the Type I error rate. An analytical strategy equivalent to this would allow us to make probability statements such as "The average implant failure at 10 years is estimated to be P_F, and in repeated sampling we would expect on $1-\alpha/2$ % of occasions that the true, unknown, value is contained within the confidence interval". This approach can be framed as a one-sample hypothesis test (*z*-test), of one proportion, see equation 1.

$$z = \frac{\widehat{P_F} - P_{BM}}{\sqrt{\frac{\widehat{P_F}(1-\widehat{P_F})}{n}}} \qquad 1$$

The temptation is to simply rearrange equation 1, solve the hypothesis test for *n,* and conclude that this is the minimal sample size required to detect if an implant is superior to the benchmark. However, this would be incorrect as this fails to recognise the uncertainty introduced by sampling variability. With small samples, sampling variability is large, and conversely when samples are large sampling variability is small. Sampling variability is one of the reason that Type I and Type II statistical errors are made. A Type I error is made when

For peer review only - http://bmjopen.bmj.com/site/about/guidelines.xhtml

we incorrectly reject a valid null hypothesis, i.e. that the true device failure rate at 10 years is equal to the benchmark. Whereas a Type II error is when the null hypothesis is incorrect, i.e. the true failure rate is not equal to the benchmark, yet we fail to reject it. One minus the Type II error rate is also known as power, see figure 1.

<Figure 1. Here>

Whilst there has been some debate with regards the use of formal sample size calculations, which are determined on the basis of power and type I errors, or by pre-specifying the desired width of the confidence interval.[12 13] We believe they are both useful tools for researchers and device manufacturers when planning clinical studies, and determining the sample sizes required to draw reliable conclusions, by explicitly stating expected power of a sample and or defining the desired width of estimated confidence interval. When planning a clinical study, decisions need to be made with regards to the uncertainty in which the null hypothesis should be rejected (commonly but not necessarily $\alpha = 0.05$), and power $(1-\beta)$ which is the ability to correctly reject the null hypothesis when it is false. Therefore, simply solving a hypothesis test for *n* fails to consider the power to detect an effect when it is truly present. A study with a sample size that gives 100% power will correctly reject the null hypothesis on all occasions (although in practice 100% power is likely impossible), and correspondingly a sample size that gives 50% power will correctly reject the null hypothesis on only half of occasions. This is the essence of nearly all superiority designed clinical studies.

Non-inferiority and benchmarking studies

However, in the context of a benchmarking system, the definition of superiority is restrictive. For example, if the true failure rate of the implant of interest is exactly equal to the benchmark ($\widehat{P_F} = P_{BM}$), it would be desirable to say that the implant has met the required standard. Furthermore, it is impossible to demonstrate that it is superior, because the standard error will always be positive and not 0, and therefore the upper confidence interval will always be greater than the benchmark ($\widehat{P_F} + \Phi(z_{1-\alpha/2})\widehat{SE}(\widehat{P_F}) > P_{BM}$). Therefore, in a superiority study design, if a prosthesis failure rate is truly equivalent to the benchmark (the null hypothesis is correct), in repeated sampling we should only incorrectly reject it on 5% of occasions if $\alpha = 0.05$. Therefore, when comparing devices to a benchmark, a non-inferiority study design including sample size calculations, analysis, and reporting maybe preferable.[14-18]

Despite the linguistic similarities between superiority and non-inferiority studies, the analysis and interpretation are different. A non-inferiority framework requires the interested parties to

For peer review only - http://bmjopen.bmj.com/site/about/guidelines.xhtml

place limits around what could be described as non-inferior i.e. a non-inferiority margin (δ). This is stating that if a device failure rate was 5%, the 95% confidence interval ranged between 4.01% and 5.99%, and that the non-inferiority margin was 1%, we would be happy to conclude that device was clinically equivalent or non-inferior, see figure 2.

Figure 2: Schematic representation of inferiority, non-inferiority and superiority studies

<Figure 2 Here>

If the failure rate was 5.5% and the confidence interval ranged between 5.25% and 5.75% we would still conclude that this device was clinically non-inferior, despite being statistically inferior, with a non-inferiority margin of 1%. The methods by which one should choose an appropriate non-inferiority margin are inherently subjective, and the risk of choosing too large a margin represent a risk of exposing patients to inferior and less efficacious products. This is opposed to a margin which is too small, which in turn limits products of similar performance being introduced to the market, both the Food Drug Administration (FDA)[19] (US) and European Medicines Agency (EMA)[20] provide extensive guidance in relation to the choice of non-inferiority margins.

Aims

The aim of this study is to investigate the sample size required at the beginning of a study to demonstrate superiority and non-inferiority of implant failure compared to an external benchmark level of performance, i.e. a one-sample non-inferiority study design in the presence of censoring, and the consequences of using three common methods of estimating failure in a simulation study.

For peer review only

Method

The simulation study will be described using an Aims, Data generating process, Method of analysis, Estimands, and Performance (ADMEP) structure.[21]

Aims

The aim of this study is to describe the sample size required to identify if a prosthetic implant has a failure rate non-inferior to an external benchmark using simple analytical solutions. In addition, we will use a simulation study to determine the power to detect superiority and non-inferiority with different sample sizes, different estimands, in the presence of a non-informative competing risk (CR) (mortality), and when the true implant failure rate is the same as the benchmark.

Analytical Sample Size Calculation of One Proportion

Using analytical solutions of a *z*-test in a non-inferiority setting (see equation 2 and 3), the sample size at five non-inferiority margins (δ) at 1, 2, …., 5% was calculated with power $(1-\beta)$ varied from 10% to 99.9%. The proportion of failures ($\widehat{P_F}$) was assumed to be equal to the benchmark (P_{BM}), 5% and the Type I error rate was set at α = 2.5% (the one tail equivalent of α =0.05).

$$z = \frac{\widehat{P_F} - P_{BM} - \delta}{\sqrt{\frac{\widehat{P_F}\left(1-\widehat{P_F}\right)}{n}}} \qquad 2$$

$$n = \widehat{P_F}\left(1-\widehat{P_F}\right)\left(\frac{z_{1-\alpha} + z_{1-\beta}}{\widehat{P_F} - P_{BM} - \delta}\right)^2 \qquad 3$$

Data Generating Process (DGP)

Two different data generating mechanisms with varying sample sizes (N= 100, 200, 400, 800, 1600, 3200, 6400) were explored: 1) implant failure with no censoring, and 2) implant failure with censoring for mortality. Implant failure and mortality data was simulated independently (non-informative censoring) from a parametric survival distribution (2 parameter Weibull) (Failure: $T{\sim}Weib(\lambda = 0.01, \gamma = 0.71)$; Mortality: $T{\sim}Weib(\lambda = 0.017, \gamma = 1.32)$, where λ and γ are scale and shape parameters in a Weibull distribution respectively.[22 23] Parameters were chosen to so failure and mortality were simulated to have 5% and 30% occurrence at ten

For peer review only - http://bmjopen.bmj.com/site/about/guidelines.xhtml

years respectively, which is similar to data from the National Joint Registry of England Wales and Northern Ireland,[24] and administratively censored at 10 years. 1000 repetitions of each DGP were simulated, assuming nominal coverage of a *z*-test was achieved Monte Carlo error would be equal to 0.7%.

Method of Analysis

We investigated the first DGP (no competing risks) using the z-test, see equation 2, where the proportion of failures is simply estimated by the number of observed failures (f) within the sample divided by the total sample.

$$\widehat{P_F} = \frac{f}{n} \qquad 4$$

A failure function, 1-$\hat{S}(t)$[25] (1-KM), approach was used to estimate failure where n_j is the number at risk of failure preceding time t_j and f_j is the number of failures at time t_j, see equation 5, with confidence intervals estimated using asymptotic variance of $log(-\log \hat{S}(t_j))$.[26]

$$\hat{S}(t) = \prod_{j|t_{j\le t}} \left(\frac{n_j - f_j}{n_j}\right) \qquad 5$$

The second DGP was similarly investigated using 3 approaches: 1) A z-test, where the proportion of failures was calculated excluding those who died prior to 10 years; 2) A failure function, 1-$\hat{S}(t)$[25] (1-KM), with the addition of censoring individuals when they died was also used. 3) A non-parametric competing risk (CR) model[27] was also explored, see equation 6. The CR model calculates the cumulative incidence for failure type *k*. It is estimated using a KM approach for all failure types at the instance before the failure of interest, $\hat{S}(t_{j-1})$, and then multiplying it by the cause specific hazard, d_{kj}/n_j, i.e those experiencing the failure of interest divided by all individuals who remain at risk.

$$\widehat{CIF}_k(t) = \sum_{j|t_{j\le t}} \hat{S}(t_{j-1}) \frac{d_{kj}}{n_j} \qquad 6$$

For peer review only - http://bmjopen.bmj.com/site/about/guidelines.xhtml

All analyses were conducted in Stata (Stata Statistical Software: Release 14.1 College Station, TX: StataCorp LP.)

Estimands

The estimand of interest was cumulative implant failure at 10 years, and its 95% confidence intervals, in a hypothetical world where implant failure is the only possible outcome.

The first DGP explicitly simulates data where implant failure is the only possibly outcome, and the analytical methods purport to estimate net failure, whereas the second DGP simulates data where there are two potential outcomes (implant failure or death). The simple proportion of failures ($\widehat{P_F}$) and failure function (1-$\hat{S}(t)$) both estimates net failure (assuming the untestable assumption of independence between the CR and implant failure). Whereas the CR approach ($\widehat{CIF}_k(t)$) estimates crude failure, which can be described as the real world probability of failure, which allows for the fact that some individuals die before their implant fails.

Performance

Performance was assessed in the superiority study setting using bias, $\frac{1}{n}\sum_{i=1}^{n}\left(\hat{P}_{F.i} - P_{BM}\right)$, root mean square error (RMSE) (absolute error), $\frac{1}{n}\sum_{i=1}^{n}\sqrt{\left(\hat{P}_{F.i} - P_{BM}\right)^2}$, power to detect superiority, i.e. a non-inferiority margin at 0, which is equal to type I error in a one tailed test, $\frac{1}{n}\sum_{i=1}^{n}100\left(\hat{P}_{F.upp.i} \leq P_{BM}\right)$, coverage of the 95% confidence interval, $\frac{1}{n}\sum_{i=1}^{n}100\left(\hat{P}_{F.low.i} \leq P_{BM} \leq \hat{P}_{F.upp.i}\right)$, and confidence interval width, $\frac{1}{n}\sum_{i=1}^{n}\left(\hat{P}_{F.upp} - \hat{P}_{F.low}\right)$. Similarly we estimated performance in a non-inferiority setting using power (1-Type II error) to detect non-inferiority at 1, 2, 3, 4 and 5% at 10 years, $\frac{1}{n}\sum_{i=1}^{n}100\left(\hat{P}_{F.upp.i} \leq P_{BM} + \delta\right)$.

For peer review only - http://bmjopen.bmj.com/site/about/guidelines.xhtml

Results

Analytical approach to sample size calculation of non-inferiority against a benchmark

Using analytic sample size calculations, non-inferiority against a benchmark sample size was calculated and is presented in Figure 3.

Figure 3. The sample size required to detect non-inferiority of the failure proportion with non-inferiority margins (δ) at 1, 2, 3, 4, and 5%.

<Figure 3 Here>

With a non-inferiority margin of 3% failure, power of 50%, 203 individuals are required at the beginning of the study, whereas with 90% power, 555 individuals are required at the beginning of the study. There is an approximately log-linear association between sample size and power between 50% and 90%, at all non-inferiority margins. However, sample size rapidly increases as the non-inferiority margin reduces.

Simulation approach to sample size calculation of superiority against a benchmark

Results from the simulation study using a superiority design are presented in Figure 4.

Figure 4: Performance characteristics of 5 analyses; *z*-test and Kaplan-Meier when there is no competing risk (NCR), and a *z*-test, KM and CIF in the presence of competing risk (CR).

<Figure 4 Here>

The method of analysis and DGP process are indicated using 5 different line styles. It is clear that the cumulative incidence function (CIF), which estimates crude failure, estimated in the presence of CR consistently underestimates net failure by 0.5%, whereas the *z*-test, an estimate of net failure, in the presence of CR overestimates failure rates. Notably, the accuracy (RMSE) in the estimates of all methods are similar, and as sample size increases they reduce. However, the CIF and *z*-test in the presence of CR are biased estimates of net failure in comparison to KM, and RMSE does not tend to zero with increasing sample sizes. Correspondingly, coverage of the 95% CI reduces as the sample size increases for both the CIF and *z*-test. The CIF power to detect superiority erroneously increases as a consequence of a consistent difference in the estimand (crude vs. net failure) and narrowing confidence intervals. The width of the estimated confidence intervals, across all methods, consistently

For peer review only - http://bmjopen.bmj.com/site/about/guidelines.xhtml

decreases as sample size increases. Despite their homogeneity, the width of the CI from the CIF is approximately 1% larger than that of the KM estimate in the presence of CR.

Simulation approach to sample size calculation of non-inferiority against a benchmark

Simulation results using a *Z*-test with no competing risks were compared with analytic sample size calculations assuming no competing risks, see supplementary figure 1. Simulation results are generally concordant with analytical results. However, analytical results tend to slightly over-estimate power when the non-inferiority margin is greater than 3%.

Results from the simulation study using a non-inferiority paradigm, are presented in Figure 5.

Figure 5: Power to detect non-inferiority at 1,2,3,4 and 5% below a 95% benchmark performance. The DGP and method of analysis are presented in separate panels. The sample size is indicated on the horizontal axis.

<Figure 5 Here>

When no competing risk is present, a 3% non-inferiority margin, and at samples sizes of 200 or 800, a *z*-test has 46% and 94% power to detect non-inferiority respectively, and KM has 34% and 91% power to detect non-inferiority. When a non-informative competing risk is present, a 3% non-inferiority margin, and sample sizes N=200 or N=800, a *z*-test has 22% & 44% power to detect non-inferiority respectively, KM has 26% and 86% power to detect non-inferiority respectively, and the CIF has 48%, and 99% power to detect non-inferiority respectively.

Mean performance estimates from the simulation study are tabulated in the supplementary table 1.

For peer review only - http://bmjopen.bmj.com/site/about/guidelines.xhtml

Discussion

This study investigates how, and how many individuals are required to demonstrate non-inferiority in the failure rate of a medical device (arthroplasty prosthesis) compared to an external benchmark in the presence or absence of a competing risk.

Net failure estimated using 1-KM provides unbiased estimates in the presence or absence of a non-informative competing risk, whereas a simple Z-test or CIF over and under estimate failure in the presence of a non-informative competing risk respectively. Whilst there is reasonable agreement between analytical and simulation estimates of the sample size required to conduct a non-inferiority study, the failure to incorporate an adjustment for censoring due to mortality lead to erroneous estimates of power.

Using 1-KM to estimate failure in the presence of a non-informative competing risk (estimating net failure), a sample size of N=1600, will ensure coverage at nominal levels, have a confidence interval width of approximately 2.3% and a RMSE of 0.46%, but have a 35%, 88%, 97%, 97%, and 97% power of demonstrating non-inferiority at margins of 1%, 2%, 3%, 4%, and 5% respectively.

This study has a number of strengths. We have shown the well-known differences between net and crude failure. Despite the persistent suggestion in arthroplasty research that 1-KM overestimates implant failure, it is clear that it is an unbiased estimate of net failure but a biased estimate of crude failure, a patient's personal chance of revision surgery. The CIF (crude failure) in a CR model unsurprisingly underestimates net failure, assuming independence between the CR and implant failure. We have demonstrated the number of individuals required at the onset of a benchmarking study for a variety of non-inferiority margins, under two different data generating process, and the width of the estimated CI. Despite the exemplar of arthroplasty, similar methods would apply to any discipline interested in conducting a formal benchmarking process.

However, this study has a number of important limitations. 1) We simulated data from a Weibull model with an uncorrelated competing risk, and whilst it provides a convenient and sensible method of generating data in an arthroplasty example, more complex models with correlated competing risks may be appropriate in other areas. 2) We assume that time to revision surgery is a reasonable outcome of interest. However, alternative outcomes, e.g. Patient Reported Outcome Measures (PROMS), could similarly be incorporated into a non-inferiority benchmarking design. 3) The threshold for revision is assumed to be homogenous

For peer review only - http://bmjopen.bmj.com/site/about/guidelines.xhtml

between different surgeons in this simulation. 4) We have not considered the effect of analysing data from multiple sources or combining pre-existing data. However, methods of meta-analysis in non-inferiority settings are well documented.[28 29] 5) The continuous accrual of data, and regular assessment of data against benchmarks of interest will inevitably lead to multiple hypothesis testing, and therefore the risk of incorrectly accepting or rejecting the null hypothesis of inferiority against the benchmark will increase. The decision to repeatedly look at the data must be made *a priori,* and due consideration for the multiple testing should be made in order to preserve the Type I error rate.[30] Related to this point is that once a prosthesis meets the benchmark level of performance, this should be considered the beginning of the monitoring process, and as new data is accrued periodic assessment should occur to ensure satisfactory performance is being maintained. 6) Current implant rating organisations consider the failure rates of individual components (i.e. head, stem, and cups separately) opposed to the implanted construct. Despite the technical difficulties of disentangling the failures of each individual component, the multiple testing that ensues will ultimately require larger samples in order to preserve the Type I error rate. 7) We have adopted a conservative scenario, where the implant failure rate is equal to the benchmark, in a more optimistic scenario, where the implant failure rate is less than the benchmark, fewer individuals will be required to detect non-inferiority. Conversely, when implant failure rate is more than the benchmark but less than the non-inferiority margin, larger samples will be required. However, unless implant manufacturers are, *a priori,* willing to propose superiority opposed to non-inferiority a conservative standpoint would appear sensible. Finally and most importantly, 8) we critically assume a simple unadjusted analysis provides a causal interpretation, and there is no residual confounding or selection bias that inhibits making a fair comparison. However, we recognise that assumption has little validity given the strong variation in implant use and failure rates between those young and old, and between males and females.[24] Therefore, the necessity to adequately describe the population used within the benchmarking process or adopt a stratified approach to benchmarking is compelling. Similarly, the current design of arthroplasty benchmarking studies, specifically the use of an external benchmark, makes more sophisticated methods of adjusting data, either via regression, weighting or matching unfeasible. Therefore, there may be a number of benefits to choosing a contemporary prosthesis combination with established performance to which all other prosthesis can be benchmarked against. We also recognise as with nearly all clinical studies and registers that enrolled or consenting participants can be substantially different from those not enrolled or those who withhold consent to be included in a register. These

For peer review only - http://bmjopen.bmj.com/site/about/guidelines.xhtml

potential differences may have a profound impact on how the results from benchmarking studies should be interpreted.

Despite some authors suggesting that a 1-KM overestimates implant survival in the presence of competing risk,[7] it is clear that this is not universally true, and it is very important to understand what is being estimated (net or crude failure) and for what purpose, recognise that implant failure rate is not necessarily equal to the rate of revision surgery in arthroplasty, and ensure analyses reflect the research question being presented.[31] The three methods of analysis, i.e. a *z*-test (simple proportion), 1-KM and the CIF can provide equivalent or subtly different estimates depending on the data generating process. When data are fully complete (DGP1), i.e. everyone is fully observed for the period of interest and there is no censoring, the failure rate estimated by a simple proportion, 1-KM and the CIF will all be identical, variability in coverage and power will be due to inherent differences in the methods used to construct confidence intervals. When there is censoring due to a competing risk (i.e. DGP2) differences in the estimates arise. These differences are best understood when data are fully observed for individuals experiencing the event of interest or the relevant competing event. A simple proportion (*z*-test) excludes patients who experience the competing event from the denominator, whereas the CIF includes them, the numerator for both methods is simply the observed number of failures at 10 years. In this scenario, it is easy to see that failure rate estimated by a simple proportion will be greater than the CIF due to the reduction in the denominator. It is also clear the CIF does not estimate the proportion of failures that would have occurred in those individuals who have experienced the competing event and therefore will underestimate the net failure rate of the device (the numerator is too small relative to the denominator). Whereas, the KM method excludes individuals from the analysis who experience the competing risk, thereby reducing the denominator, which results in a higher failure rate than the CIF.

This balancing act is very well known and demonstrated by the seminal work of Gooley et al.[32]. They generally recommend that researchers report the CIF, but similarly, note that if one is interested in evaluating a cause-specific failure the CIF may be misleading, and inferences should be made from functions which are based solely on the hazard of failure from the cause of interest, i.e. use the KM estimator. Putter et al.[8] similarly state that the “naive Kaplan–Meier estimator describes what would happen if the competing event could be

For peer review only - http://bmjopen.bmj.com/site/about/guidelines.xhtml

prevented to occur, creating an imaginary world in which an individual remains at risk of failure from the event of interest" i.e. an immortal patient cohort. Ranstam et al.[31] describe this in an arthroplasty setting as the "implicit assumption that the patient will be alive until the implant fails". This is not to say the CIF (crude failure) is not of interest to arthroplasty surgeons, patients, regulators or healthcare planners. It is just to say the CIF (crude failure) estimates a different quantity, which reflects the real word (observed) probability of implant failure (arthroplasty revision rate), whilst acknowledging the CR of mortality explicitly prevents failure from the cause of interest. If the CIF was used in a benchmarking setting, this may have a number of different consequences: 1) it could lead to a number of implants being claimed as non-inferior to the benchmark, when they are in fact inferior, due to differences between crude and net failure, and 2) the method of estimation may lead to the selective introduction of implants in older patients where mortality is higher, which is likely to reduce the reported failure rate.

The broad success of arthroplasty, and currently used benchmark failure rate of 5% at 10 years, necessitates the use of small non-inferiority margins. A small non-inferiority margin of 1% in absolute risk represents a 20% increase in relative risk of failure compared to a benchmark of 5%. The minimum numbers of individuals required to demonstrate non-inferiority of a device where its true failure rate is equal to the benchmark is unsurprisingly large. Modest sample sizes (n=1600) have limited power (35%) to detect non-inferiority, and only when sample sizes become large (n=6400) does power increase substantially (90%).

Despite differences in the performance of estimators and interpretation of estimands, the choice of sample size at the beginning of a study should be based on the desire to obtain sufficiently precise estimates (small RMSE and narrow CI's) which mitigate Type II errors for a given non-inferiority margin which is tolerable to the public, surgeons, and regulators. Whereas, the choice of sample size that should remain at risk at the end of the benchmarking period is somewhat more difficult to determine. Two possible reasons to ensure the numbers at risk at the end of the period are large include; 1) maintaining the performance and minimising the width of confidence intervals and 2) ensuring sufficient numbers of patients experience 10 years of the risk of revision for the estimates and the benchmarking process to be credible.

From a conservative perspective, it is simple to request that the same number of patients for a given level of power, $(1-\beta)$, and non-inferiority margin, δ, at the beginning of the

For peer review only - http://bmjopen.bmj.com/site/about/guidelines.xhtml

benchmarking period, remain at risk at the end of the benchmarking period. However, this ignores that censoring due to mortality is expected to occur in an elderly populations, and will delay the benchmarking process. Whereas, it would not be difficult to request a specified delay (i.e. 10 years) from surgery on the jth patient until the benchmarking assessment is made, a process which is similar to pre-registration of randomised trials. This will implicitly allow for censoring due to mortality, without pre-specifying how much censoring will occur, and assumes there is no loss to follow-up (which is often assumed in arthroplasty registers).

Conclusion

The choice of non-inferiority margin, initial sample size, or desired width of the confidence interval in a benchmarking study are all subjective decisions, and can only be chosen by balancing the risk of incorrectly awarding a benchmarking standard to an implant with a failure rate beyond the non-inferiority margin, vs benchmark inflation where all devices receive benchmarks and the entire process lacks credibility. However, this study clearly demonstrates how 1-KM provides unbiased estimates of net implant failure, in a conservative scenario when the failure rate of an implant being tested is equal to the benchmark and has a competing risk which is uncorrelated to the event of interest.

For peer review only

Figures

Figure 1: Simple hypothesis testing framework and illustration of Type I and II errors, power and true negatives.

Figure 2: Schematic illustration of a non-inferiority testing paradigm.

Figure 3: Analytical estimates of sample sizes at varying levels of power and a fixed alpha (5%) from a one-sample non-inferiority design using a Wald test of one proportion at 5 non-inferiority margins (δ) at 1%, 2%, 3%, 4%, and 5%.

Figure 4: Performance characteristics of 3 analysis methods, under 2 data generating mechanisms. Performance measures include Type I error, Bias, Root Mean Square Error (RMSE), Nominal coverage of 95% Confidence Interval (Coverage), and Confidence Interval width (CI width).

Figure 5: Power to detect non-inferiority at 1,2,3,4 and 5% below a 95% benchmark performance. The DGP and method of analysis are presented in separate panels. The sample size is indicated on the horizontal axis.

For peer review only

References

1. View of a global benchmarking system and International Working group progress. International Society of Athroplasty Registries; 2016; Manchester.
2. Kandala NB, Connock M, Pulikottil-Jacob R, et al. Setting benchmark revision rates for total hip replacement: analysis of registry evidence. *BMJ* 2015;350:h756. doi: 10.1136/bmj.h756
3. National Institue of Clinical Excelence. Total hip replacement and resurfacing arthroplasty for end-stage arthritis of the hip, 2014.
4. NHS Supply Chain. Orthopaedic Device Evaluation Panel 2016 [Available from: https://www.supplychain.nhs.uk/odep/.
5. Australian Goverment Department of Health. Prostheses List - Guide to listing and setting benefits for prostheses 2015 [Available from: http://www.health.gov.au/internet/main/publishing.nsf/Content/health-privatehealth-prostheseslist.htm.
6. Nederlandse Orthopaedische Vereniging. Classification Orthopaedic Implants: NOV Total Hip Prosthesis 2016

2016 [Available from: http://www.orthopeden.org/uploads/ky/In/kyInjf44QX2oW1qhk8PKRA/HIPLIST-NOV-2016.pdf.
7. Fennema P, Lubsen J. Survival analysis in total joint replacement: an alternative method of accounting for the presence of competing risk. *J Bone Joint Surg Br* 2010;92(5):701-6. doi: 10.1302/0301-620X.92B5.23470
8. Putter H, Fiocco M, Geskus RB. Tutorial in biostatistics: competing risks and multi-state models. *Stat Med* 2007;26(11):2389-430. doi: 10.1002/sim.2712
9. Lambert PC, Dickman PW, Nelson CP, et al. Estimating the crude probability of death due to cancer and other causes using relative survival models. *Stat Med* 2010;29(7-8):885-95. doi: 10.1002/sim.3762
10. Gardner MJ, Altman DG. Confidence-Intervals Rather Than P-Values - Estimation Rather Than Hypothesis-Testing. *Brit Med J* 1986;292(6522):746-50.
11. Sterne JA, Davey Smith G. Sifting the evidence-what's wrong with significance tests? *BMJ* 2001;322(7280):226-31.
12. Bland JM. The tyranny of power: is there a better way to calculate sample size? *BMJ* 2009;339:b3985. doi: 10.1136/bmj.b3985
13. Metcalfe C. Statistical power: still an essential element in sample size calculation. Response to:The tyranny of power: is there a better way to calculate sample size? *BMJ* 2010;339:b3985. doi: 10.1136/bmj.b3985
14. D'Agostino RB, Massaro JM, Sullivan LM. Non-inferiority trials: design concepts and issues - the encounters of academic consultants in statistics. *Stat Med* 2003;22(2):169-86. doi: 10.1002/sim.1425
15. Fleming TR. Current issues in non-inferiority trials. *Stat Med* 2008;27(3):317-32. doi: 10.1002/sim.2855
16. Koch GG. Comments on 'Current issues in non-inferiority trials' by Thomas R. Fleming, Statistics in Medicine, DOI: 10.1002/sim.2855. *Stat Med* 2008;27(3):333-42. doi: 10.1002/sim.2923
17. Sedgwick P. What is a non-inferiority trial? *Bmj-Brit Med J* 2013;347 doi: ARTN f6853

10.1136/bmj.f6853

For peer review only - http://bmjopen.bmj.com/site/about/guidelines.xhtml

18. Piaggio G, Elbourne DR, Pocock SJ, et al. Reporting of noninferiority and equivalence randomized trials: extension of the CONSORT 2010 statement. *JAMA* 2012;308(24):2594-604. doi: 10.1001/jama.2012.87802
19. U.S. Department of Health and Human Services, Food and Drug Administration, Center for Drug Evaluation and Research (CDER), et al. Non-Inferiority Clinical Trials to Establish EffectivenessGuidance for Industry 2016 [Available from: https://www.fda.gov/downloads/Drugs/Guidances/UCM202140.pdf accessed 05/04/2017.
20. European Medicines Agency (COMMITTEE FOR MEDICINAL PRODUCTS FOR HUMAN USE). GUIDELINE ON THE CHOICE OF THE NON-INFERIORITY MARGIN [EMEA/CPMP/EWP/2158/99] 2006 [Available from: http://www.ema.europa.eu/docs/en_GB/document_library/Scientific_guideline/2009/09/WC500003636.pdf accessed 05/04/2017.
21. Morris T, White I, Crowther MJ. Using simulation studies to evaluate statistical methods. Unpublished teaching material, 2015.
22. Crowther MJ, Lambert PC. Simulating complex survival data. *Stata J* 2012;12(4):674-87.
23. Crowther MJ, Lambert PC. Simulating biologically plausible complex survival data. *Stat Med* 2013;32(23):4118-34. doi: 10.1002/sim.5823
24. National Joint Registry. 13th Annual Report 2016. Hemel Hempstead2016.
25. Kaplan EL, Meier P. Nonparametric-Estimation from Incomplete Observations. *J Am Stat Assoc* 1958;53(282):457-81. doi: Doi 10.2307/2281868
26. Kalbfleisch JD, Prentice RL. The Statistical Analysis of Failure Time Data. 2nd ed ed. New York: Wiley 2002.
27. Coviello V, Boggess M. Cumulative incidence estimation in the presence of competing risks. *The Stata Journal* 2004;4(2):103-11.
28. Crowther MJ, Langan D, Sutton AJ. Graphical augmentations to the funnel plot to assess the impact of a new study on an existing meta-analysis. *Stata J* 2012;12(4):605-22.
29. Sutton AJ, Cooper NJ, Jones DR, et al. Evidence-based sample size calculations based upon updated meta-analysis. *Stat Med* 2007;26(12):2479-500. doi: 10.1002/sim.2704
30. Pocock SJ. Clinical Trials: A practical approach. Chichester: John Wiley & Sons 1983:147-154.
31. Ranstam J, Karrholm J, Pulkkinen P, et al. Statistical analysis of arthroplasty data. II. Guidelines. *Acta Orthop* 2011;82(3):258-67. doi: 10.3109/17453674.2011.588863
32. Gooley TA, Leisenring W, Crowley J, et al. Estimation of failure probabilities in the presence of competing risks: new representations of old estimators. *Stat Med* 1999;18(6):695-706.

For peer review only - http://bmjopen.bmj.com/site/about/guidelines.xhtml

The truth

		H_0 is True	H_0 is False
Decision with observed data	Reject H_0:	Type I Error (α)	True positives or power ($1-\beta$)
	Fail to reject H_0:	True negatives	Type II Error (β)

H_0= Null hypothesis, H_1= alternate hypothesis

H_0 & H_1 in a non-inferiority benchmarking setting.

H_0: Device failure rate is greater than the benchmark

H_1: Device failure rate is less than or equal to the benchmark

Figure 1: Simple hypothesis testing framework and illustration of Type I and II errors, power and true negatives.

147x104mm (300 x 300 DPI)

iew only

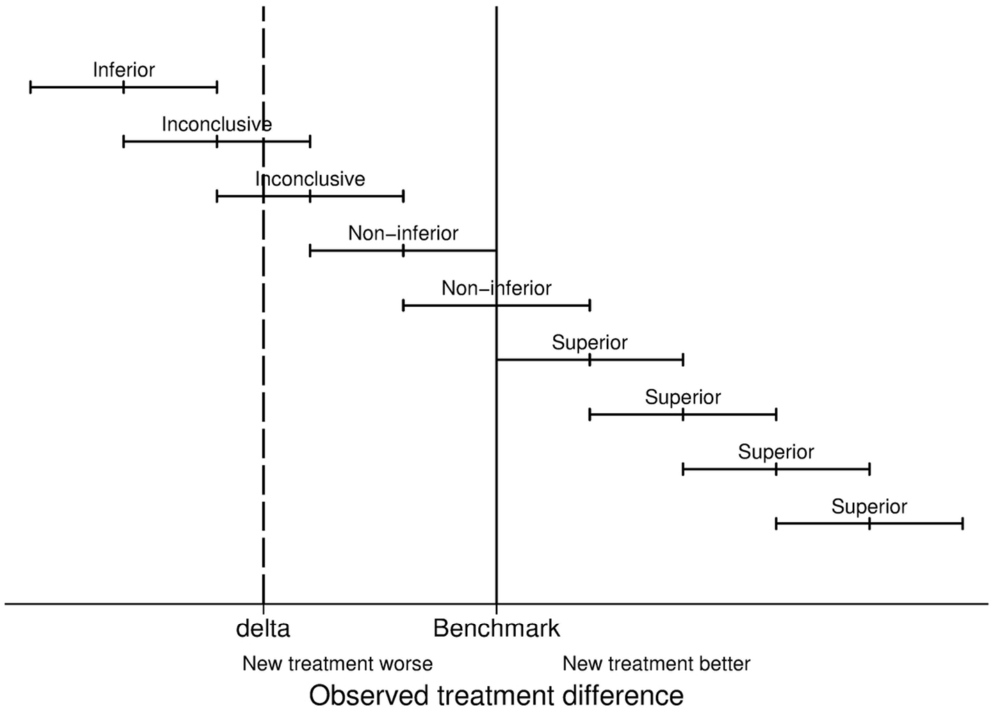

Figure 2: Schematic representation of inferiority, non-inferiority and superiority studies

101x73mm (300 x 300 DPI)

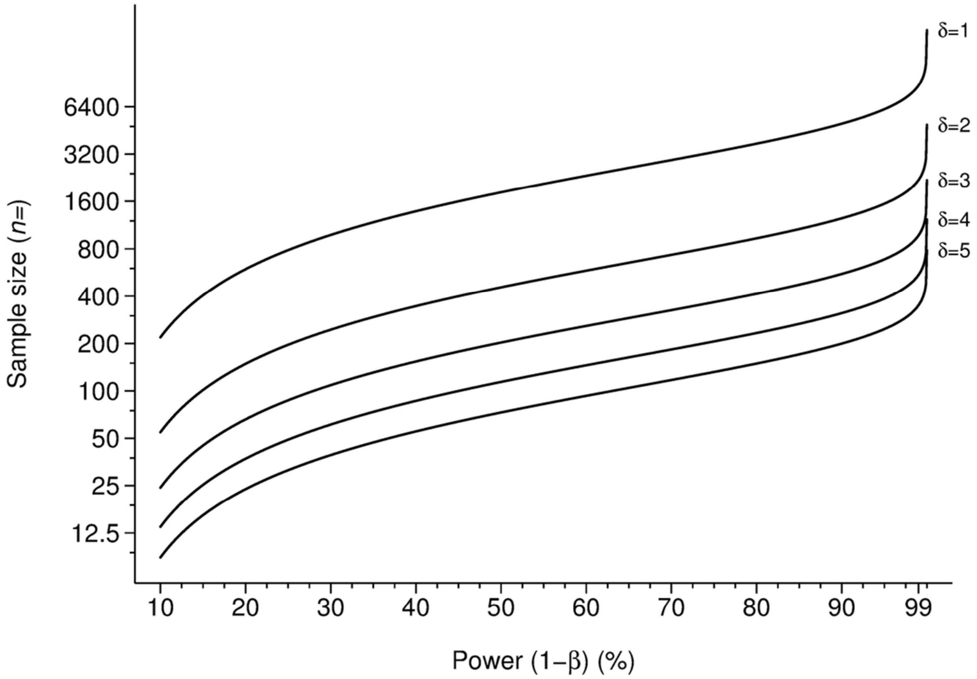

Figure 3. Sample size required to detect non-inferiority of the failure proportion with non-inferiority margins (δ) at 1, 2, 3, 4, and 5%.

101x73mm (300 x 300 DPI)

For peer review only - http://bmjopen.bmj.com/site/about/guidelines.xhtml

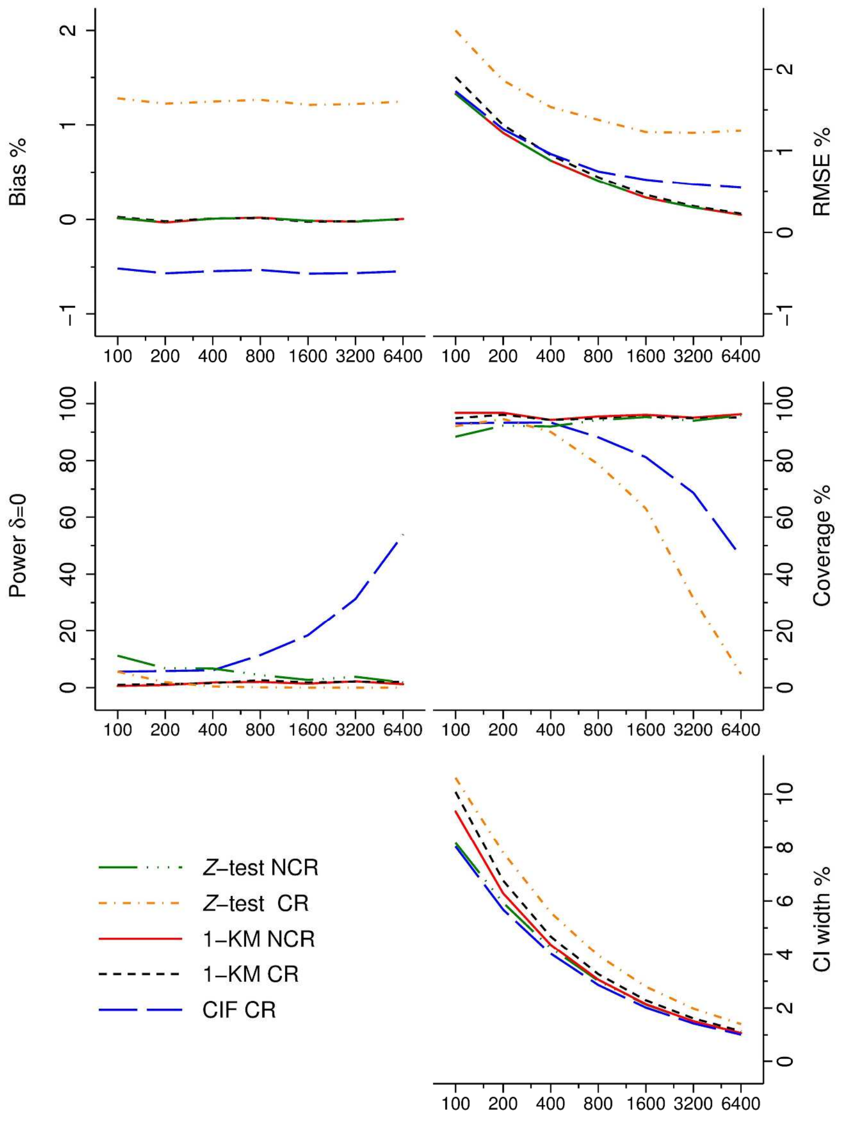

Figure 4: Performance characteristics of 3 analysis methods, under 2 data generating mechanisms. Performance measures include Type I error, Bias, Root Mean Square Error (RMSE), Nominal coverage of 95% Confidence Interval (Coverage), and Confidence Interval width (CI width).

254x317mm (300 x 300 DPI)

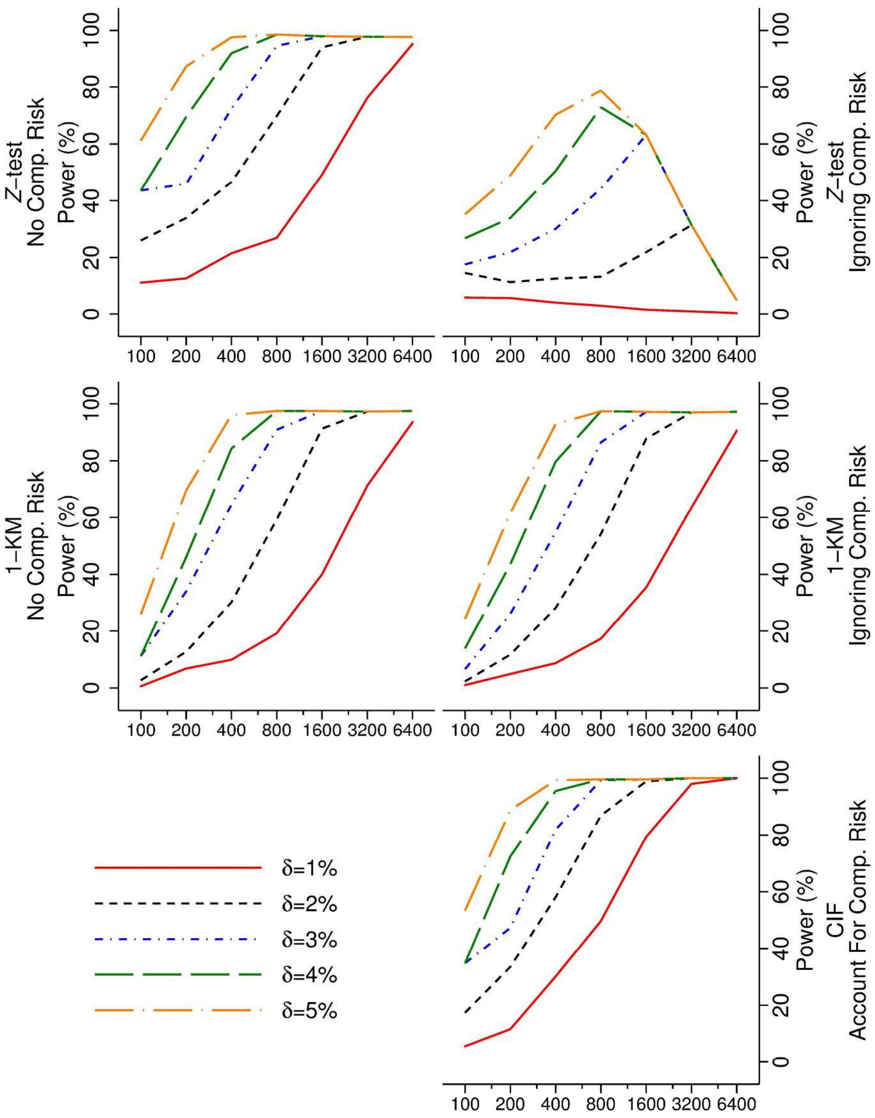

Figure 5: Power to detect non-inferiority at 1,2,3,4 and 5% below a 95% benchmark performance. The DGP and method of analysis are presented in separate panels. The sample size is indicated on the horizontal axis.

254x317mm (300 x 300 DPI)

Supplementary Table 1. Tabulation of performance metrics of simulation results.

Method	N	Bias	RMSE	Coverage	95% CI Width	Power (1-β) to Detect Non-Inferiority (%) δ=0	δ=1	δ=2	δ=3	δ=4	δ=5
	100	0.01	1.70	96.8	9.35	0.6	0.6	2.7	11.2	11.2	26
	200	-0.03	1.22	96.8	6.29	0.9	6.8	12.7	34	46	69.6
	400	0.01	0.88	94.3	4.36	1.8	9.9	30.2	64.6	84.3	96.1
KM NCR	800	0.02	0.63	95.5	3.06	2	19.2	59.3	90.9	97.5	97.5
	1600	-0.01	0.43	96.1	2.14	1.4	40	91.3	97.5	97.5	97.5
	3200	-0.02	0.31	95.1	1.51	2.2	71.3	97.3	97.3	97.3	97.3
	6400	0.01	0.22	96.3	1.07	1.2	93.6	97.5	97.5	97.5	97.5
	100	0.03	1.90	94.9	10.08	1	1	2.3	6.6	13.9	24.4
	200	-0.02	1.32	96.1	6.76	1.2	4.9	11.7	26.1	43.6	61.6
	400	0.01	0.95	94.3	4.68	1.6	8.7	28.2	54.8	79.7	92.9
KM CR	800	0.02	0.68	94.8	3.26	2.6	17.3	54.2	86.5	97.4	97.4
	1600	-0.02	0.46	95.4	2.29	1.8	35.4	87.8	97.2	97.2	97.2
	3200	-0.02	0.32	94.9	1.61	2.1	63.6	97	97	97	97
	6400	0.00	0.23	95.2	1.14	2	90.6	97.2	97.2	97.2	97.2
	100	-0.52	1.73	93.1	8.04	5.6	5.6	17.4	34.8	34.8	53.4
	200	-0.57	1.27	93.3	5.68	5.8	11.6	33.5	47.3	72.4	88.8
	400	-0.54	0.96	93.4	4.03	6.1	30.1	58.1	81.9	95.5	99.3
CIF CR	800	-0.53	0.75	88.2	2.86	11.4	49.7	86.9	99.3	99.6	99.6
	1600	-0.57	0.65	81.2	2.02	18.4	79.4	98.9	99.6	99.6	99.6
	3200	-0.56	0.59	68.7	1.43	31.3	98	100	100	100	100
	6400	-0.55	0.55	46.1	1.01	53.9	100	100	100	100	100
	100	0.01	1.70	88.4	8.17	11.2	11.2	26	43.6	43.6	61.2
	200	-0.03	1.22	92.3	5.94	6.8	12.7	33.9	45.9	69.5	87.4
	400	0.01	0.88	92	4.25	6.7	21.5	46.6	72.4	92	97.6
Z-test NCR	800	0.02	0.63	94.2	3.02	4.4	26.9	69.8	94.6	98.6	98.6
	1600	-0.01	0.43	95.3	2.13	2.7	49.2	94.1	98	98	98
	3200	-0.02	0.31	94	1.51	3.8	76.2	97.8	97.8	97.8	97.8
	6400	0.01	0.22	95.9	1.07	1.8	95.3	97.7	97.7	97.7	97.7
	100	1.28	2.47	92.1	10.61	5.6	5.8	14.6	17.6	26.8	35.2
	200	1.22	1.86	94.6	7.81	1.9	5.6	11.4	21.9	33.9	49
	400	1.25	1.53	90.1	5.57	0.4	4	12.6	30.1	50.5	70.3
Z-test CR	800	1.27	1.38	78.7	3.96	0.1	2.9	13.3	44.2	72.9	78.8
	1600	1.21	1.23	63.2	2.80	0	1.5	21.9	63.2	63.2	63.2
	3200	1.22	1.22	31.4	1.98	0	0.9	31.4	31.4	31.4	31.4
	6400	1.25	1.25	4.8	1.40	0	0.3	4.8	4.8	4.8	4.8

KM= Kaplan-Meier ; CIF= Cumulative Incidence Function; NCR=No Competing Risks ; CR= Competing Risks ; δ =Non-inferiority margin

For peer review only

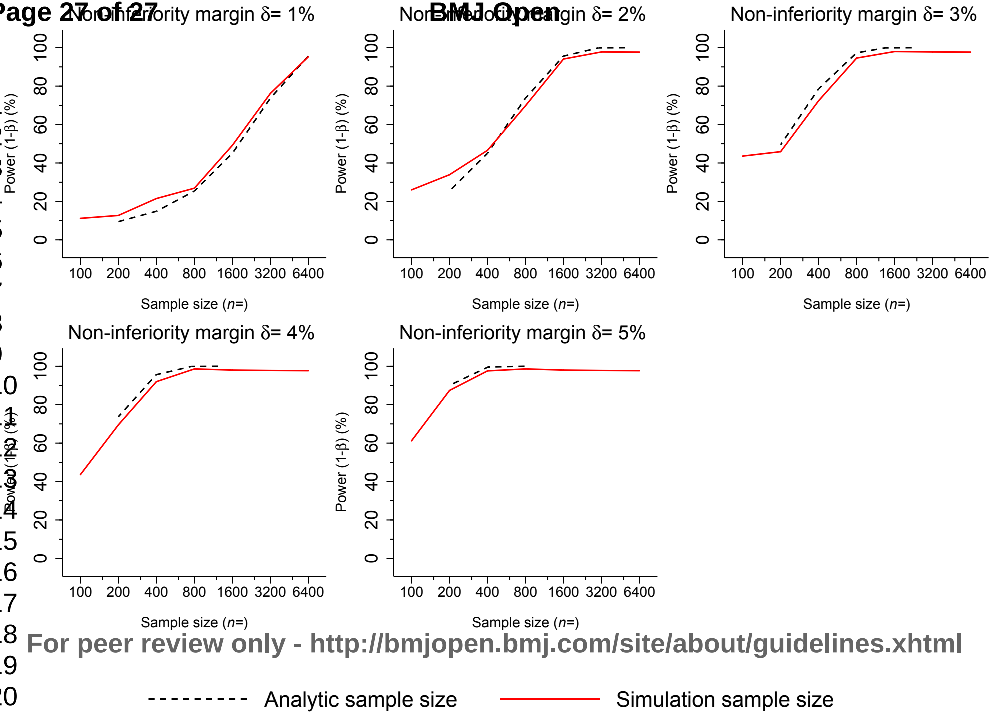

For peer review only - http://bmjopen.bmj.com/site/about/guidelines.xhtml
